# Odyssey of environmental and microbial interventions in maize crop improvement

**DOI:** 10.3389/fpls.2024.1428475

**Published:** 2025-01-09

**Authors:** Alok Kumar Singh, Alok Kumar Srivastava, Parul Johri, Manish Dwivedi, Radhey Shyam Kaushal, Mala Trivedi, Tarun Kumar Upadhyay, Nadiyah M. Alabdallah, Irfan Ahmad, Mohd Saeed, Sorabh Lakhanpal

**Affiliations:** ^1^ Indian Council of Agriculture Research (ICAR) – National Bureau of Agriculturally Important Microorganism, Mau, Uttar Pradesh, India; ^2^ Amity Institute of Biotechnology, Amity University Uttar Pradesh, Lucknow, India; ^3^ Department of Biotechnology, Dr. Ambedkar Institute of Technology for Divyangjan (AITH), Kanpur, Uttar Pradesh, India; ^4^ Department of Life Sciences, Parul Institute of Applied Sciences and Research and Development Cell, Parul University, Vadodara, Gujarat, India; ^5^ Department of Biology, College of Science, Imam Abdulrahman Bin Faisal University, Dammam, Saudi Arabia; ^6^ Basic & Applied Scientific Research Centre, Imam Abdulrahman Bin Faisal University, Dammam, Saudi Arabia; ^7^ Department of Clinical Laboratory Sciences, College of Applied Medical Science, King Khalid University, Abha, Saudi Arabia; ^8^ Department of Biology, College of Sciences, University of Hail, Hail, Saudi Arabia; ^9^ School of Pharmaceutical Sciences, Lovely Professional University, Phagwara, Punjab, India

**Keywords:** abiotic and biotic stress, breeding improvement, maize, PGPR, transgenic

## Abstract

Maize (*Zea mays*) is India’s third-largest grain crop, serving as a primary food source for at least 30% of the population and sustaining 900 million impoverished people globally. The growing human population has led to an increasing demand for maize grains. However, maize cultivation faces significant challenges due to a variety of environmental factors, including both biotic and abiotic stresses. Abiotic stresses such as salinity, extreme temperatures, and drought, along with biotic factors like bacterial, fungal, and viral infections, have drastically reduced maize production and grain quality worldwide. The interaction between these stresses is complex; for instance, abiotic stress can heighten a plant’s susceptibility to pathogens, while an overabundance of pests can exacerbate the plant’s response to environmental stress. Given the complexity of these interactions, comprehensive studies are crucial for understanding how the simultaneous presence of biotic and abiotic stresses affects crop productivity. Despite the importance of this issue, there is a lack of comprehensive data on how these stress combinations impact maize in key agricultural regions. This review focuses on developing abiotic stress-tolerant maize varieties, which will be essential for maintaining crop yields in the future. One promising approach involves the use of Plant Growth-Promoting Rhizobacteria (PGPR), soil bacteria that colonize the rhizosphere and interact with plant tissues. Scientists are increasingly exploring microbial strategies to enhance maize’s resistance to both biotic and abiotic stresses. Throughout the cultivation process, insect pests and microorganisms pose significant threats to maize, diminishing both the quantity and quality of the grain. Among the various factors causing maize degradation, insects are the most prevalent, followed by fungal infections. The review also delves into the latest advancements in applying beneficial rhizobacteria across different agroecosystems, highlighting current trends and offering insights into future developments under both normal and stress conditions.

## 1 Introduction

Maize (*Zea mays* L.) is India’s third most significant grain crop, trailing behind wheat and rice. It plays a key role as a food source for billions of people in both advanced and developing nations (Canton, 2021). It serves as a staple food for millions of people living in poverty. On a global scale, maize is one of the most widely cultivated crops, covering more than 100 million hectares in various countries (Tubiello et al., 2009). The extensive cultivation of maize is essential for the livelihoods of millions of small-scale farmers. With the global population expected to increase from approximately 7.7 billion in 2020 to around 9.3 billion by 2050, demand for maize in developing countries is projected to double over this period (Nur et al., 2020). The increasing human population and rising demand for animal-based products are driving up maize consumption. However, expanding maize production is challenged by limited arable land and various biotic and abiotic stressors that affect yield, productivity, and quality. To tackle these challenges, scientists have genetically engineered maize by introducing specific genes, leading to transgenic varieties with improved traits. The release of the first commercially available transgenic maize in the United States in 1996 marked a significant milestone in crop genetic modification. Today, maize is the most extensively modified crop in terms of genetic engineering, with the highest number of transgenic varieties. Consequently, developing transgenic maize has become a forefront strategy for enhancing the genetic potential of this essential crop (Raza et al., 2019).

Environmental factors are critical in influencing maize crop yield. Maize productivity and traits are affected by both their genetic makeup and the environmental conditions they face (Hudson et al., 2022). Plants naturally progress through different stages to complete their life cycle. However, recent changes in climate, such as irregular precipitation and temperature fluctuations, have created significant challenges. These shifts have caused extended droughts and temperature variations that fall outside optimal ranges (Yadava et al., 2017; Odell et al., 2022), affecting crop production. In India, maize is cultivated in a range of environments, from semi-arid to sub-humid and moderate climates. Climate change is a persistent and critical issue that is continuously reshaping the world. It has already caused significant changes and is expected to drive even more substantial global transformations in the future. While crop productivity has improved over the past two decades, the growing vulnerability of plants to abiotic stresses presents a new challenge in sustaining high yields amidst shifting climate patterns (Mao et al., 2015; Alotaibi, 2023). Developing crops with tolerance to abiotic stress may be vital for sustaining crop yields in the future (Duvick, 2005). In reaction to various environmental stresses, plant cells activate complex signaling pathways that involve hormones, transcription factors, and signal transducers. These signals coordinate to regulate stress-responsive genes, resulting in the production of proteins and enzymes that help plants manage stress (Zandalinas et al., 2018). Maize production is facing significant threats from variable drought conditions, high temperatures, and inconsistent rainfall (Lobell et al., 2014). Consequently, current research is focused on enhancing traits that confer tolerance to abiotic stresses. However, identifying the genetic factors responsible for this tolerance remains a difficult task (Mao et al., 2015). Abiotic stress tolerance is determined by complex quantitative traits that are frequently associated with other developmental features. These traits are controlled by numerous quantitative trait loci (QTL), each having a minor impact on the overall trait expression, which complicates their identification and modification (Miao et al., 2017). This study seeks to evaluate the effects of various abiotic stresses on maize productivity.

Using plant-supporting microorganisms such as arbuscular mycorrhizal (AM) fungi, various other beneficial fungi, and plant growth-promoting bacteria (PGPB) presents a promising alternative strategy (Dodd and Ruiz-Lozano, 2012; Barea, 2015). These microorganisms can enhance crop yields without the need for additional mineral nitrogen. Research on maize has highlighted their substantial benefits, including improved growth and better crop quality (Montañez et al., 2012; Berta et al., 2014; Dhawi et al., 2015; Marks et al., 2015; Dartora et al., 2016). Employing PGPR is a promising strategy to mitigate the environmental effects of chemical fertilizers, pesticides, and herbicides, as well as to address abiotic stress. According to Kloepper (1978), PGPR are soil bacteria that colonize the plant rhizosphere and support growth through various mechanisms. With the increasing demand for agricultural output to support a growing global population, excessive reliance on chemical inputs has often led to soil degradation (Bhusal et al., 2021). Plant growth-promoting microbes have been shown to boost plant nutrition and reduce the need for pesticides (Kimotho et al., 2019). With modern agriculture grappling with environmental and social issues from industrialization and the pressure to feed a growing population, PGPR presents a viable solution for sustaining high yields while reducing environmental impact (Pérez-Montaño et al., 2013).

Researchers are focusing more on microbial strategies to boost maize’s resistance to biotic and abiotic stresses. Maize faces damage from insect pests and microorganisms during both pre-harvest and post-harvest periods, which reduces its quality and yield (Burlakoti et al., 2024). Maize plants and grains are affected by various pathogenic bacteria and insects, leading to an estimated annual global loss of 9.4%. Insects are the main cause of maize degradation and reduced yields, with fungi contributing as a secondary factor (Khosravi et al., 2007; Sitara and Akhter, 2007). Maize cultivation is significantly challenged by pests, with insect and soil pests posing some of the most serious threats. According to Roberts (2006), global annual losses from plant diseases, encompassing both direct and indirect effects, are estimated to reach nearly $40 billion.

This review explores how microorganisms can boost maize yield and development. Microbial and transgenic methods, including PGPR, help alleviate abiotic stresses. Some microorganisms enhance plant growth directly, independent of pathogen presence, while others offer indirect benefits by safeguarding the plant from soil-borne diseases.

## 2 Factors affecting maize production

**Figure 1:**
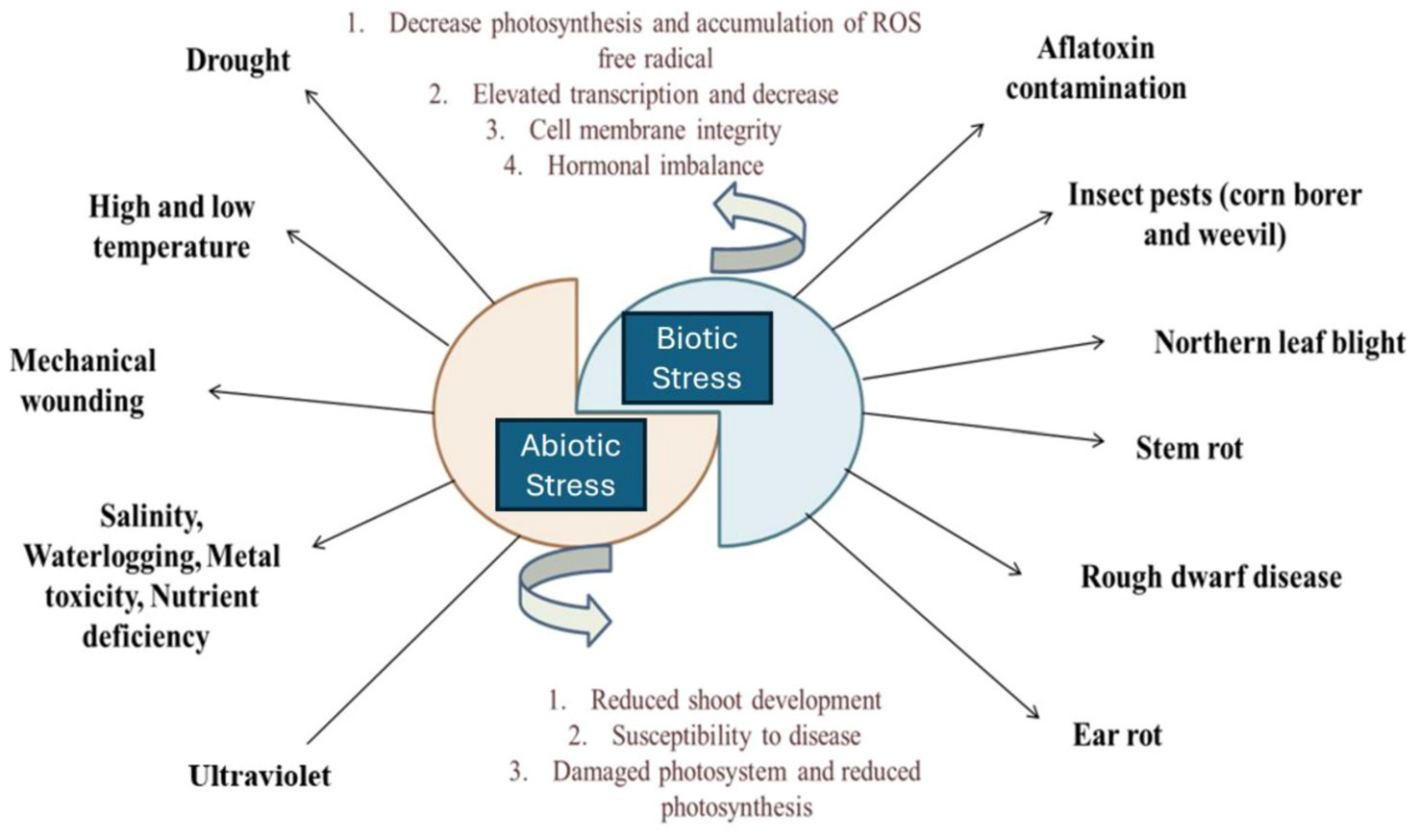
Several disease and disorders of maize plant through biotic and biotic stress.

Abiotic and biotic factors are major environmental influences on crop productivity. Rising global temperatures exacerbate these factors, worsening climate change. Abiotic stressors such as cold, drought, heat, salinity, and flooding negatively impact plant growth and productivity, causing various physiological, morphological, molecular, and biochemical changes (**Figure 1**) (Popp et al., 2013). These stressors also affect soil conditions by altering soil composition, pH, and its physicochemical and biological properties. Biotic factors encompass beneficial organisms such as decomposers, natural enemies, and pollinators, as well as pests including weeds, diseases, arthropods, vertebrates, and human-induced influences (Burlakoti et al., 2024).

### 2.1 Abiotic stress

Abiotic factors such as temperature, drought, cold and salinity, negatively impact various stages of plant development. These stresses are complex, affecting various aspects of plant function at molecular, physiological, and cellular levels, including grain filling, maturation, and flowering (Atkinson and Urwin, 2012). Plant breeders and researchers have consistently focused on enhancing maize’s resilience to significant abiotic stressors, such as flooding, salt, drought, and severe temperatures, as doing so is essential for preserving yield stability (Halford et al., 2015). With the global population increasing and environmental conditions changing rapidly, the development of crop cultivars with stress tolerance is desperately needed. Understanding how plants respond to multiple stresses at a molecular level is vital for developing crops with broad-spectrum stress tolerance. Consequently, it is crucial to comprehend the dynamics of abiotic stress tolerance and to develop innovative strategies to counteract its negative effects on agriculture. Recent advancements in bioinformatics, including genome-wide association studies, gene mining, functional omics, and research into transcription factors and microbial interactions, offer promising methods for enhancing maize’s resilience to abiotic stress (Takeda and Matsuoka, 2008).

Abiotic stresses, like waterlogging, cold, nitrogen deficiency, salinity, and drought, significantly affect maize growth and yield. For instance, salinity stress disrupts maize’s biochemical and physiological processes, leading to cellular imbalances, ionic disturbances, impaired nitrogen fixation and respiration, and the inhibition of crucial metabolic enzymes involved in photosynthesis (Iqbal et al., 2020). One serious stressor that can drastically lower maize yields across the globe is drought. Its effects change according to the stress’s length, intensity, and growth stage (Kim and Lee, 2023). Drought affects kernel weight from the silking stage through to maturity. Maize plants have evolved a number of mechanisms to deal with drought, and certain germplasms exhibit traits that improve drought resilience. In severe drought conditions, maize production can decrease by 20-30% due to rapid leaf wilting and reduced photosynthesis during grain filling (Qiao et al., 2024). Recent advancements in microbial techniques are being investigated to enhance grain selection and development, aiming to boost drought tolerance (Sade et al., 2018).

Plants have evolved several adaptation mechanisms to optimize water usage and continue growing in harsh environments (Yuriko et al., 2014). Extreme light conditions, whether excessively low or high, can disrupt physiological processes and negatively impact plant development. For instance, too much light can lead to photo-oxidation, which generates reactive oxygen species that damage biomolecules and enzymes, ultimately reducing plant productivity (Li et al., 2010). Both freeze temperatures and extreme heat contribute to crop losses (Koini et al., 2009; Pareek et al., 2010Soil conditions such as alkalinity, acidity, and salinity (Bromham et al., 2013; Bui, 2013), along with pollutants and human activities (Emamverdian et al., 2015), adversely impact plant growth and yield. Acidic soils affect nutrient availability, leading to deficiencies and disrupting normal growth patterns (Rorison, 1986). Early exposure to salinity can cause ion toxicity and osmotic imbalance, with prolonged stress worsening these effects. Plants respond to salinity by avoiding high-salinity areas, excreting excess ions through their roots, or compartmentalizing ions to minimize damage to critical cellular functions (Silva et al., 2010). In cold conditions, plants may avoid freezing by preventing tissue supercooling or developing resistance to freezing. Some species achieve this through “cold acclimation,” adapting to cold temperatures by enhancing their anti-freezing responses during shorter daylight periods (Thomashow, 2010).

### 2.2 Maize physiological disorder due to abiotic stress

Abiotic stresses affecting maize growth include water shortages (drought), excessive moisture (waterlogging), extreme temperatures (both high and low), salt stress, and nutrient deficiencies.

#### 2.2.1 Drought stress

Maize, a crop highly sensitive to drought, is grown in a variety of climates, from semi-arid to temperate zone, including drought-prone areas in Africa, the Europe, Asia, and Americas (Xie et al., 2017). Maize may reduce its life cycle to prevent stress in order to escape drought, especially during critical reproductive periods. Drought is recognized as a major environmental challenge, drawing significant attention from environmentalists and agricultural researchers (Burlakoti et al., 2024). It poses a critical agricultural issue that affects plant growth and yields worldwide, impacting nearly all major agricultural regions and having extensive socio-economic consequences (Kumari et al., 2004; Xie et al., 2017). During drought conditions, stress response genes and various growth parameters are negatively affected. Reduced water availability compromises membrane integrity, reduces cell size and produces reactive oxygen species, which speeds up the aging of leaves and the loss of agricultural productivity (Osmolovskaya et al., 2018; Muhammad et al., 2023). Significant physiological and molecular changes occur in plants when they lack water. These changes include decreased photosynthesis, damaged photosynthetic machinery, increased ethylene production, and changed chlorophyll levels (Basu et al., 2016). Drought stress adversely affects critical physiological processes in maize, such as photosynthesis, water relations, and nutrient uptake. It induces oxidative stress by increasing the production of reactive oxygen species (ROS), which can damage cellular components and impair growth (Cai et al., 2020). This response is essential for maintaining water use efficiency during periods of water scarcity. Additionally, drought stress leads to oxidative stress characterized by the overproduction of ROS, which can damage cellular structures and impair physiological functions (Agunbiade and Babalola, 2023). Drought stress causes an accumulation of free radicals, disrupting membrane functions and protein structures, leading to lipid peroxidation and cell death. With climate change intensifying these conditions, the frequency and severity of droughts are expected to increase (Sharma et al., 2023). The precise molecular mechanisms behind reproductive drought sensitivity are not yet fully understood due to the intricate regulation of drought stress by various genes. A more thorough understanding of the molecular biology and connections in reproductive drought tolerance is essential for developing next-generation maize cultivars that are both climate-smart and drought-resistant. Despite considerable research over recent decades to elucidate drought tolerance mechanisms in maize, improving drought resilience through traditional breeding methods has been challenging because drought characteristics are complicated and multigenic. To overcome this, advanced genome analysis, breeding strategies, and molecular genetics tools, genes that improve drought tolerance in maize can be found and exploited using tools like CRISPR-Cas (Singh et al., 2023).

#### 2.2.2 Temperature stress

High temperature stress is a critical factor contributing to reduced maize productivity globally. Research indicates that temperatures exceeding 35°C can negatively affect maize development, particularly during critical growth stages such as anthesis and grain filling. For example, temperatures above 40°C during these stages can lead to severe yield losses (Waqas et al., 2021). Heat stress disrupts several physiological processes, including membrane integrity, photosynthesis, and respiration. The over-accumulation of ROS under heat stress can cause oxidative damage, leading to cell toxicity and impaired plant functions (Tiwari and Yadav, 2019). Maize plants exhibit a considerable decrease in net photosynthesis when leaf temperatures rise beyond 38°C; the fall is more pronounced when temperatures rise abruptly as opposed to gradually (Magar et al., 2019). This decrease in photosynthesis is not because of stomatal closure, as transpiration rates increase with temperature. Instead, temperatures above 32.5°C reduce Rubisco’s active state, leading to its complete inactivation at 45°C. Additionally, higher leaf temperatures cause a decrease in 3-phosphoglyceric acid levels. Rubisco activation, which is associated with the expression of a new activase polypeptide, adjusts to rising temperatures. Crafts-Brandner and Salvucci suggest that Rubisco inactivation is the main factor limiting net photosynthesis at temperatures above 30°C (Scafaro et al., 2023).

Chilling injury mainly affects maize leaves, causing premature senescence. Low temperatures (around 10°C) coupled with intense light significantly impair the irreversible suppression of photosynthesis caused by CO2 assimilation (Foyer et al., 2012; Riva-Roveda et al., 2016). A study by Janda et al. (1999) found that treating young maize seedlings in a hydroponic setup with salicylic acid provided defence against the stress of low temperatures. By decreasing catalase activity, this treatment improved the seedlings’ resilience to freezing by increasing the activity of antioxidant enzymes including glutathione reductase and peroxidases (Alam et al., 2022).

Global warming is anticipated to cause more frequent extreme temperature events, resulting in both hotter and colder days. Such temperature extremes can severely impact maize germination, seedling growth, and overall productivity (Zaidi et al., 2023). In northern China, the risk of high-temperature stress is increasing, while in the US, maize yields can drop significantly when temperatures exceed 29-30°C. To address these challenges, developing new crop management strategies or pursuing selective breeding may be necessary (Bhusal et al., 2021).

#### 2.2.3 Combined temperature and drought stress

Hussain et al. (2019) found substantial decreases in a number of plant development metrics, including as fresh and dry shoot weight, leaf area, kernels per ear, 100-kernel weight, and grain yield per plant, in their investigation of the effects of heat and drought stress on maize hybrids. These reductions were more pronounced under the combined effects of heat and drought stress, with drought stress having a more severe impact than heat stress alone. Heat stress notably affected chlorophyll concentration, while drought stress significantly reduced relative water content, a parameter unaffected by heat stress alone. Additionally, drought stress, especially when combined with heat stress, led to elevated intercellular carbon dioxide concentrations. Bhusal et al. (2021) noted that transpiration rates varied with stress conditions: dropping further under combined heat and drought stress, decreasing under drought stress, and increasing under heat stress.

Under stress conditions, the total antioxidant capacity (T-AOC) significantly increased compared to normal growth conditions. heat shock proteins, free proteins and Soluble sugars, increased in response to drought and combined heat and drought stress, while soluble protein concentrations reduced. Heat stress alone did not impact nitrogen levels in the roots, leaves, or stems, but drought stress significantly reduced nitrogen concentrations in the leaves. Neither heat nor drought stress alone caused substantial reductions in phosphorus and potassium levels compared to normal conditions. However, when heat and drought stresses were combined, there was a notable decrease in nitrogen levels in the roots, as well as reductions in phosphorus and potassium levels across the leaves, roots and stems (Hussain et al., 2019).

#### 2.2.4 Salinity stress

Salinity stress is a significant abiotic factor that adversely affects maize (*Zea mays*) growth and productivity, leading to various physiological disorders. High salt concentrations in the soil create osmotic stress, which limits water uptake and results in ion-specific toxicity, particularly from sodium ions. This combination of stressors can severely hinder maize’s physiological processes, including photosynthesis, respiration, and nutrient uptake, ultimately reducing plant growth and yield (Wang et al., 2020). Research indicates that maize is particularly sensitive to salinity, with marked reductions in growth observed in saline soils (Ngara et al., 2012). Maize can tolerate salt stress up to 3 dS m−1; however, above this level, the plants experience significant osmotic stress-related physiological changes, nutrient imbalances, and ion toxicity. These changes include stunted growth, shorter internodes, and reductions in leaf potassium, magnesium, and phosphorus, with maize shoots being more sensitive to salt stress than roots. Various factors, such as crop growth stages, genetic traits, and soil conditions, all contribute to reduced production under salt stress (Islam et al., 2024). Vennam et al. (2024) report that maize plants modify their metabolic processes, biochemical, and physiological, in response to salt stress through a variety of pathways. Osmotic stress results from salt reducing the soil water potential, which hinders the plants’ ability to take in water and nutrients. The presence of cations like sodium (Na+), potassium (K+), and calcium (Ca2+), as well as anions like chloride (Cl−) and nitrate (NO3−), leads to soil salinity, which is frequently brought on by insufficient rainfall or soil deterioration (Zorb et al., 2009). The physicochemical and biological balance of the soil, as well as crop yield, seed germination, and nutrient and water uptake, are all impacted by salinity stress (Farooq et al., 2015). Additionally, it has a detrimental effect on the nodulation process, which lowers crop yields by reducing nitrogen fixation. Because the nitrogenase enzyme involved in this process is less active in salinized environments, nitrogen fixation—which is crucial for plant growth is especially vulnerable to salt stress. Moreover, excessive salt within plant cells becomes poisonous and inhibits growth, and high soil salinity reduces the amount of water absorbed by roots. Salinity’s osmotic and ion-toxic effects not only hinder plant growth but also interfere with soil microorganisms’ ability to function. Extreme osmotic conditions in the soil prevent both plants and microorganisms from efficiently absorbing water, and fungi are more susceptible to high salt concentrations than bacteria (Ying et al., 2020).

#### 2.2.5 Mechanical wounding

Mechanical injury, whether resulting from herbivore attacks or abiotic factors like wind, rain, and hail, serves as the primary entry point for pathogen infection. In addition to the injured tissues, this damage also causes metabolic reactions in the plant’s unaffected sections. In response to a localized injury, signal transduction pathways are triggered throughout the plant, inducing defence systems and promoting healing. Researchers have explored maize’s response to wounding at the injury sites and the related signal transduction pathways that extend to other regions of the plant using phosphoproteomic and molecular techniques (Schilmiller and Howe, 2005). A recent comparative transcriptome analysis revealed that mechanical wounding leads to the differential expression of 407 genes in maize, with 134 being upregulated and 273 downregulated. The upregulated genes are involved in protein synthesis, phytohormone signaling, and responses to various stresses, while downregulated genes are associated with primary metabolism and developmental processes (Kumari et al., 2023).

#### 2.2.6 Waterlogging

Waterlogging is a significant abiotic stress that adversely affects maize, leading to various physiological disorders and reduced yield. When maize is subjected to waterlogging, it experiences a decline in leaf greenness due to chlorosis, which is linked to a decrease in chlorophyll content (Tian et al., 2019). This stress also negatively impacts plant height, leaf number and root development, ultimately resulting in reduced biomass accumulation (Huang et al., 2022). Furthermore, waterlogging at critical growth stages, such as flowering, can severely impair ear development and grain yield (Kaur et al., 2021). According to Zaidi et al. (2010), there is insufficient oxygen reaching the roots due to the quick consumption and delayed diffusion of oxygen. Oxygen is a vital component for plant existence. In China and other countries where waxy maize is a major crop, waterlogging often occurs during the jointing stage, causing substantial yield losses. Understanding the physiological mechanisms of waterlogging stress during this stage is vital for formulating strategies to lessen its impact. The threshold for waterlogging stress in waxy maize typically occurs between 4 to 6 days during the jointing stage. Waterlogging during this period significantly reduces yield, primarily by decreasing kernel numbers by 6.7-15.5%, which leads to a 9.9-20.2% reduction in final yield. Additionally, waterlogging poses a serious risk to the development of waxy maize production as it hinders the synthesis of photosynthetic sources and the movement of photo assimilates, especially during the summer (Zhang et al., 2023).

#### 2.2.7 Ultraviolet

Excessive UV-B radiation can drastically affect crop yields. Under normal sunlight conditions, plants mitigate UV-B damage to macromolecules through repair or replacement mechanisms. However, unpredictable surges in UV-B exposure, often due to periodic ozone layer depletion, can overwhelm these natural defenses (Casati et al., 2011b). As the protective function of the ozone layer weakens, plants especially sessile species like maize—must significantly enhance their resistance to intense UV-B radiation. This challenge is particularly severe for high-altitude maize landraces, which must adapt to these increased UV-B levels in addition to their usual acclimatization to standard UV-B fluences (Casati et al., 2011a).

#### 2.2.8 Nutrient inadequacy

To complete their life cycle, Plants need a minimum of 14 vital mineral nutrients. Unfortunately, natural soils often lack these critical elements in quantities sufficient to support optimal plant growth (Liang et al., 2023). Nitrogen, a crucial macronutrient, is particularly important because it is fundamental to the synthesis of proteins, chlorophyll, and nucleic acids are example of secondary and primary organic components found in plants (Xu et al., 2012). A shortage of nitrogen can seriously impede the growth of maize, resulting in greatly decreased yields (Liang et al., 2013).

#### 2.2.9 Metals stress

Heavy metal pollution in soil, caused by ongoing industrial processes, intensive agriculture, and other human actions, poses significant environmental challenges. These metals, with densities exceeding 4 g/cm³, are toxic even at low concentrations and are non-degradable (Rahman et al., 2023). Heavy metal toxicity can lead to various physiological disorders in maize, including reduced growth, impaired photosynthesis, and nutrient imbalances. For instance, copper (Cu) toxicity has been shown to cause severe symptoms in maize, such as stunted growth, leaf chlorosis, and root length inhibition (Franco et al., 2023). Addressing this pollution is essential for safeguarding ecosystems, as traditional remediation methods are often expensive and harmful to soil health. According to Shen et al. (2013), a workable and affordable substitute for heavy metal removal from soil is phytoremediation, which involves using plants and the microorganisms they are linked with to remove the metals. The inclusion of bacteria in phytoremediation processes can further enhance its effectiveness. Microbes, being highly sensitive to heavy metal stress, are valuable indicators of contamination. The increasing interest in leveraging microbial diversity for heavy metal remediation underscores its low cost, environmental safety, and adaptability, making it a promising solution (Wang and Chen, 2013).

## 3 To maintain the productivity of maize through different approaches for breeding

The International Institute of Tropical Agriculture (IITA) focuses its maize breeding program on two main objectives: improving populations to create open-pollinated varieties (OPVs) and developing inbred lines to produce superior hybrids. To achieve these goals, various strategies are employed, each tailored to specific aims. Population improvement can involve enhancing genetic traits within a single population through methods like mass or family selection, or improving traits across populations using recurrent selection techniques. A critical initial step in any breeding program is selecting the appropriate germplasm for both the pool and the population. Breeders of maize can access a variety of genetic variation sources, and new developments in genomics are driving the creation of breeding methods and decision support systems that optimize the effective utilization of these genetic resources (Warburton et al., 2008).

Genomic strategies for enhancing maize start during the pre-breeding phase by leveraging the extensive genetic diversity of landraces and exotic germplasm. At this stage, markers are mainly employed for Analysis of genetic diversity and characterisation, which helps identify heterotic groups and clusters to guide cross-breeding strategies for population development. Genomic tools facilitate the detection of genetic variations in landraces, which can then be incorporated into adapted germplasm. Advances in next-generation genotyping, along with improvements in data management and informatics, have opened up new possibilities for modern breeding techniques (Gorjanc et al., 2016). The rapid advancement of Advanced sequencing technologies with high throughput have greatly reduced sequencing costs, allowing for a comprehensive evaluation of breeding lines and germplasm accessions. This progress enhances genotype-based selection and prediction, leading to improvements in maize productivity, nutritional quality, and resilience, and enabling the development of diverse breeding strategies. To complement these advancements, precise phenotyping methods and high-throughput techniques are being refined to maximize genetic gains and accelerate cultivar improvement. Managing the extensive volumes of meta-data, phenotypic and genotypic, generated requires advanced systems for data capture, storage, and integration. Additionally, innovative methods for handling and analyzing large-scale data are crucial for interpreting molecular breeding results. Integrating traditional and genomics-assisted breeding approaches demands advanced information and communication technologies, as well as robust statistical analysis (Wang et al., 2020).

Understanding the genetic structure of complex quantitative traits and evaluating germplasm sets are crucial for effectively modifying beneficial alleles and genes in genomics. In the Maize Improvement Program (MIP), molecular markers are utilized throughout various stages of the breeding cycle. These markers aid in germplasm characterization, parentage validation, gene mapping for important traits, line purity and genetic identity verification, and population enhancement through recurrent selection. This technology supports germplasm improvement, enhances grain quality, develops resistance to abiotic and biotic stresses, and ensures rigorous quality control and monitor of maize varieties (Hu et al., 2018).

### 3.1 Improvement of germplasm

#### 3.1.1 Analysis and grouping of diverse germplasm

To achieve significant progress in genetic improvement, it is essential to have a deep understanding of germplasm diversity and the relationships about elite genotypes. A key initial step in breeding is classifying inserted lines into heterotic groups in order to maximize the production of synthetics and hybrids with high yields. Breeders commonly use general combining ability (GCA), specific combining ability (SCA), and other methods to select the most suitable parent lines for crossing (Gedil and Menkir, 2019). Since the advent of molecular markers, identifying unique parental lines for crossing and classifying genotypes into heterotic groups have relied on analyzing genetic diversity. In the 1990s, genetic diversity was typically assessed and genotypes categorized based on markers such as SSR, AFLP, and RAPD. However, the use of these older markers has declined with the development of techniques for high-throughput genotyping like genotyping by sequencing (GBS). The Maize Improvement Program (MIP) research team employs a diverse array of germplasm, including various maturity groups and traits from wild relatives like *Zea diploperennis*. To develop hybrids with desirable characteristics, they classify inbred lines into distinct, potentially complementary groups using a range of techniques, including testcross performance, phenological traits, and pedigree information (Gedil and Menkir, 2019).

Mengesha et al. (2017) found that traits such as heat tolerance, drought tolerance, and low soil nitrogen tolerance can be effectively combined with Striga tolerance (Meseka et al., 2018). Mendel (2017) used single nucleotide polymorphism (SNP) markers to analyze 128 lines for drought tolerance and Striga resistance, assessing their genetic diversity. This analysis helped in selecting appropriate inbred lines for hybrid formation by evaluating their genetic variability. A more precise characterization of heterotic groups can be achieved by integrating genotypic analysis and combining ability with pedigree data (Badu-Apraku et al., 2016). According to Badu-Apraku et al. (2018), markers are often used to differentiate heterotic groups in medium-to-late maturity as well as two maturity groups: early and extra-early. This approach complements traditional methods of classifying inbred lines according to phenotypic features, which are essential for developing synthetic varieties and heterotic populations (Adebayo et al., 2015).

#### 3.1.2 Identifying and using a diverse source of germplasm for genetic improvement

**Figure 2:**
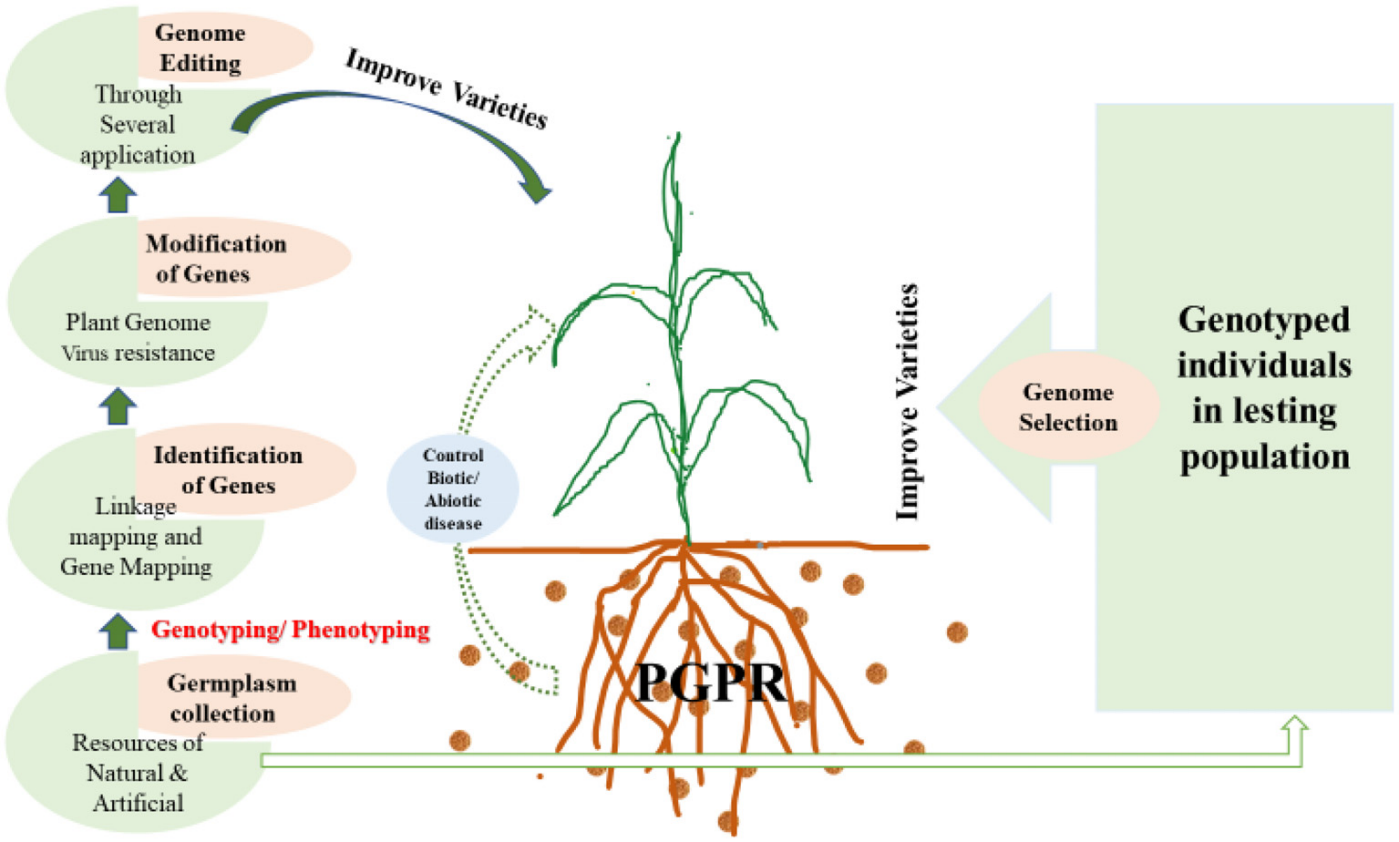
Several genomics application for improving maize variety.

Using foreign germplasm to enhance economic traits like yield is becoming increasingly common. However, integrating exotic germplasm into tropical varieties presents challenges, such as poor adaptation to tropical conditions and heightened susceptibility to unfamiliar diseases and pests. Despite these difficulties, there are notable successes where foreign germplasm has improved hybrid vigor. The MIP has adopted various strategies to boost genetic diversity in maize, as shown in **Figure 2**. For instance, genetic resources from landraces, advanced elite lines, and wild relatives have been efficiently used in DNA-based genetic characterisation and diversity evaluations (Menkir et al., 2006; Menkir and Ingelbrecht, 2007). Backcrossing with marker assistance is a more effective way to introducing rare, novel traits from wild relatives compared to traditional backcrossing, achieving results in a shorter timeframe. By incorporating wild relatives and exotic germplasm, MIP has successfully developed tropical varieties with nutrient-rich grains (Menkir et al., 2015) and resistance to parasitic weeds.

#### 3.1.3 Maize ideotype for higher maize production

An “ideotype” is a particular combination of morphological, physiological, or genetic characteristics intended to maximize crop performance in a particular application, management system, and environment (Martre et al., 2015). For advancing maize and ensuring sustainable grain production, developing an ideotype that enhances stress tolerance, adapts to high-density planting, and improves water and nutrient (NPK) uptake efficiency is essential (**Table 1**). Recent research indicates that an ideal maize ideotype would include elite shoot characteristics such as efficient kernel dehydration, rapid kernel filling, and a small leaf angle, alongside root traits like a “steep, efficient, and deep” root system. Under drought conditions, this ideotype would feature a reduced live larger cortical cell, fewer cortical cell files, and cortical area. larger cortical cells. To achieve this, Transgenic maize will be modified by adding stress-related genes to increase tolerance to biotic and abiotic challenges and boost yields. This ideotype aims to improve water and nutrient absorption, particularly in dry or nutrient-poor soils, allow for higher plant densities per unit area, and facilitate mechanical harvesting. To increase production across various agricultural settings, the breeding program has been flexible, employing techniques such as single-cross hybrid breeding, three-way cross, composite breeding, and double cross (Gong et al., 2015).

**Table 1:** NPK solubilization genes from microbial strains on maize.

Microbes (PGP)	PGP Traits	Gene of particular traits	Function	References
**Nitrogen fixing microbes-** *Bacillus aerius, Bacillus licheniformis, Bacillus subtilis, Bacillus mucilaginous, Bacillus amyloliquefaciens, etc.*	N	*Ni*f (L, R, A, H, D, K, E, N, B, Q, U, S, V, M, F, J, and Z)	*nif* genes increase the production of root nodules, plant cell expansions	Barman et al., 2017.
**Posphorus solubilisation-** *Aspergillus niger, Achromobacter xylosoxidans, Acinetobacter calcoaceticus, Aeromonas hydrophila, Arthroderma cuniculi, Acinetobacter baumannii, Bacillus (aerius, amyloliquefaciens, licheniformis, mucilaginous, subtilis, megaterium, altitudinis, thuringenesis), Burkholderia cepacia, Sphingomonas paucimobilis, Serratia nematodiphila, Serratia marcescens, Pseudomonas (stutzeri, simiae, entomophila, luteola), Paenibacillus taichungensis, Enterococcus casseliflavus, Enterococcus gallinarum*, and *Lecanicillium psalliotae, Fusarium proliferatum*	P	*Pqq* (F, A, C, E, B, and D)	*Pqq genes play a critical role in enhancing maize and other plants’ ability to absorb phosphorus into their shoots and grains.*	Puente et al., 2004.
**Potassium solubilisation-** *Aspergillus niger* and *Bacillus* sp.	K	*Trk H* and *yvlC*	*Plant stress tolerance and salt tolerance are developed by the action of Trk genes.*	Subhashini, 2015.

## 4 Microorganisms based comprehensive approaches

### 4.1 Transgenic

Tissue culture, gene transfer methods, and transgenic recombinant DNA technology are essential for developing transgenic plants. A crucial part of this process involves integrating novel features into the DNA of the host plant. Common gene transfer methods include: (i) the Agrobacterium-mediated method, which is an indirect gene transfer technique, (ii) the particle bombardment method, which is a direct gene transfer approach, and (iii) protoplast transfer technology (Ishida et al., 1996; Adams et al., 1999). While Agrobacterium-mediated transformation and particle bombardment were invented at about the same time, Agrobacterium-mediated transformation is still the most popular and simple technique. However, it has limitations, particularly with crops that are resistant to Agrobacterium. To address these challenges, particle bombardment and protoplast transfer technologies are employed, each with its own set of difficulties. Particle bombardment requires specialized equipment, while protoplast transfer demands skilled handling (Hansen and Wright, 1999). Choosing the appropriate promoter and selective marker is essential for successful gene integration. In the creation of transgenic plants, the Cauliflower mosaic virus 35S RNA constitutive promoter is frequently employed (Turrini et al., 2015). Additionally, some selection markers function as toxins that target specific organisms and are excreted by the plant’s roots. Thus, it’s critical to assess how these genetic alterations affect soil and rhizosphere bacteria (Turrini et al., 2004; Icoz and Stotzky, 2008).

Maize, first domesticated in Mesoamerica around 12,000 years ago, has become the most widely grown crop in the Americas. The US is the global leader in maize production, with South Africa, China, and Brazil, also making substantial contributions. Merely 2.5 percent of maize grown in the United States is eaten by people; the rest is fed to animals. In 2007 about 29% of U.S. maize production was dedicated to biofuels, a percentage expected to increase (Gould et al., 1991). Key pests impacting maize in the U.S. and Canada include the western corn rootworm (WCR; *Diabrotica virgifera*) and the European corn borer (ECB; *Ostrinia nubilalis*). The WCR alone causes approximately $1 billion in damage annually in the U.S., with $800 million attributed to yield losses and $200 million to pest management. ECB larvae damage maize stalks, while WCR larvae target the roots, complicating traditional pest control methods. The number of hectares planted with genetically modified maize had increased to over 35 million by 2008, following the introduction of Bt maize in 1996 to combat ECB (James, 2003).

In 2003, Bt maize engineered to resist the western corn rootworm (WCR) was introduced, targeting larvae that are highly susceptible to these genetically modified plants. The total yearly expenses of yield losses in the United States and pest control associated with corn rootworms are estimated at $1 billion (Metcalf, 1986). Prior to the commercial release of this transgenic maize, it was projected that farmers could save between $14 and $69 million annually. The new hybrid maize varieties, offering resistance to multiple insects, are expected to provide even greater financial benefits. Additionally, Bt maize has indirectly reduced the infections by pathogen (Munkvold and Desjardins, 1997). Insects feeding on plants can create wounds that increase the risk of infection by mycotoxin-producing fungi or other microbes. These fungal infections not only diminish crop yields but also cause health risks to animals and humans, and reduce the crop’s market value. The estimated annual savings from reduced fungal damage due to Bt maize are approximately $17 million (Wu et al., 2004).

There are now 21 commercially accessible varieties of maize that incorporate the cry gene. Some of these varieties are engineered with multiple traits, combining two or more cry genes with herbicide tolerance genes. Bt maize lines featuring Cry3Bb1 or Cry34/Cry35A genes provide protection against the western corn rootworm (WCR) and similar pests, demonstrating significant resistance to both lepidopteran and coleopteran pests. A prominent example is SmartStaxTM, a hybrid maize with eight transgenes, including Cry2Ab, Cry1A.105, Cry1F, Cry3Bb1, Cry34, and Cry35Ab1, along with two herbicide tolerance genes. Approved by the U.S. Environmental Protection Agency (EPA) and the Canadian Food Inspection Agency, SmartStaxTM was first commercially planted in 2010. Initially, Bt maize was primarily developed to target lepidopteran pests like the European corn borer (ECB), leading to focused research on its effectiveness against this insect. While Bt maize has proven effective in controlling ECB, factors such as location, climate, planting time, and pesticide use can influence infestation levels. Farmers benefit from Bt maize by experiencing reduced labor requirements and decreased use of toxic pesticides A recent study found that agricultural income from Bt maize increased by over $8 billion during its first 13 years of commercialization (1996-2008) (Brookes and Barfoot, 2010). Farmers with little resources have embraced Bt white maize extensively in South Africa, where it is an essential food supply. Considering its introduction in 2001, Bt maize has grown to represent two-thirds of the 1.5 million hectares of white corn cultivated in South Africa as of 2009 (James, 2003; Brookes and Barfoot, 2010).

### 4.2 Abiotic stress

#### 4.2.1 Managing drought tress in maize

In environments with limited water, microorganisms resistant to drought can promote the growth and development of maize. These microorganisms that live in the soil help plants adapt to drought by affecting trait selection and reducing the negative impacts of abiotic stress.

**Figure 3:**
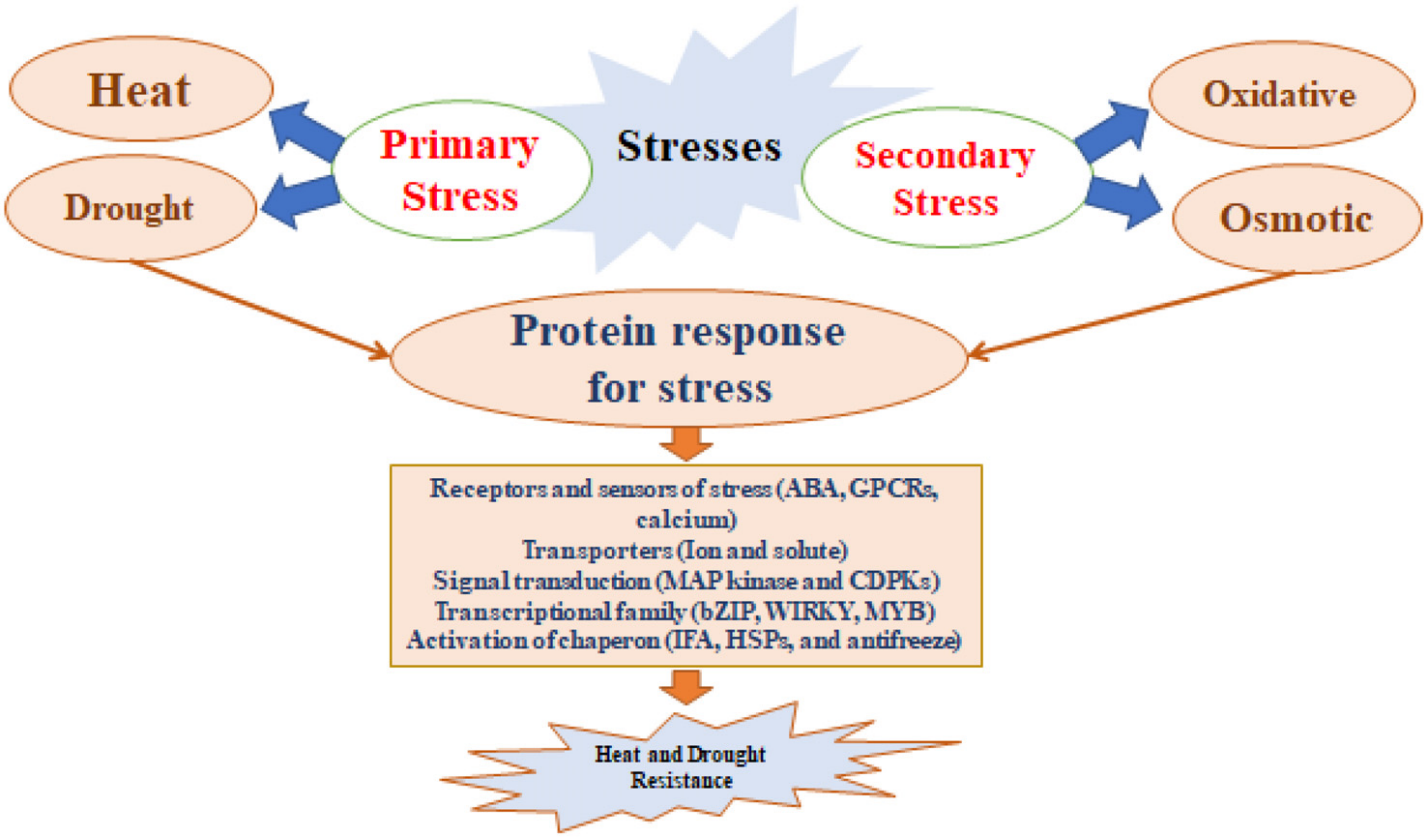
Heat and drought are first sensed via membrane-localized stress.

These beneficial microorganisms alleviate plant stress by a variety of direct and indirect methods. They influence root morphology, enzyme activity (such as ACC deaminase), osmolyte accumulation, exopolysaccharide (EPS) production, and antioxidant defenses. They also impact phytohormonal activity by producing volatile compounds, abscisic acid (ABA), indole-3-acetic acid (IAA), and cytokinins. The concept of “induced systemic tolerance” (IST) describes how microbes induce chemical and physical changes in plants, enhancing their resistance to abiotic stresses (Yang et al., 2009). These microbes can develop protective thick walls, enter a dormant state, accumulate osmolytes, and synthesize exopolysaccharides, as illustrated in **Figure 3**. In a number of ways, they lessen the negative impacts of drought on plants and soil (Farooq et al., 2009). By maintaining favorable environmental conditions and supplying essential nutrients, these microorganisms support ongoing plant growth even with limited water availability. Additionally, under stress conditions, Hormones that promote plant growth and cell division can be produced by PGPR. A potent auxin called IAA controls root differentiation, shoot growth, and cell division in response to drought stress (Farooq et al., 2009). ABA, another crucial growth regulator, increases in concentration in plants treated with PGPR, aiding in drought stress management by adjusting root water uptake andmodulating expression of gene associated with drought resistance (Jiang et al., 2013). *Azospirillum lipoferum* enhances drought tolerance in corn through ABA production and gibberellins (Cohen et al., 2009). Similarly, *Azospirillum brasilense* helps *Arabidopsis thaliana* cope with drought by raising ABA levels (Cohen et al., 2015). During drought conditions, the enzyme ACC deaminase from these bacteria converts 1-aminocyclopropane-1-carboxylate (ACC), a precursor of ethylene, into ammonia and alpha-ketobutyrate, thereby reducing ethylene levels (Bal et al., 2013). ABA, a stress hormone produced in response to cellular dehydration, plays a key role in regulating water loss by controlling stomatal closure and stress signaling pathways (Gupta and Kaushal, 2015; Yamaguchi-Shinozaki and Shinozaki, 1994).

PGPR increase drought tolerance by increasing biomass, improving water potential, and reducing water loss. They also decrease antioxidant activity while boosting levels of free amino acids, sugar production, and proline in plants (Vardharajula et al., 2011). For instance, in soybeans, drought-induced chlorophyll loss impairs photosynthesis. This issue is lessened by inoculating with Pseudomonas putida H-2-3, which raises biomass, shoot length, and chlorophyll content (Castaldini et al., 2005). In maize, inoculation with *Azospirillum brasilense* improves both relative and absolute water content during drought compared to non-inoculated plants. This treatment also enhances proline accumulation in leaves, foliar area, biomass, and root growth, with more pronounced effects observed under a 75% water reduction compared to a 50% reduction (Casanovas et al., 2002).

Combining endophytic and rhizospheric PGPR can enhance a plant’s resilience to stress. These microorganisms produce exopolysaccharides that boost drought resistance. For instance, inoculating maize with *Proteus penneri* (Pp1), and *Alcaligenes faecalis* (AF3), *Pseudomonas aeruginosa* (Pa2), has been demonstrated to increase the plant’s relative water content, as well as its protein and sugar levels (Naseem and Bano, 2014). To manage drought, bacteria employ a variety of biochemical, molecular strategies, and physiological, including the production of exopolysaccharides, compatible solutes, and spores. Exopolysaccharides help retain water during drought stress (Sandhya et al., 2009). Compatible solutes such as proline, betaine, glycine, and betaine accumulate in response to drought, aiding bacteria in maintaining membrane integrity, enzyme function, and protein stability. The impacts of drought can be lessened by combining mycorrhizal inoculation with certain bacteria, which can further improve plant growth, nitrogen intake, and water content. For instance, by raising proline levels in both shoots and roots, the combination of *Bacillus thuringiensis* and *Pseudomonas putida* reduces electrolyte leakage and stromal conductivity (Ortiz et al., 2015). Overall, microbial communities that boost drought tolerance play an important role in helping plant continue to grow and survive in water-scarce environments.

#### 4.2.2 Manage salinity stress in maize

Due to the fact that the accumulation of Na and Cl ions can upset the balance of nutrients, negatively affecting plant development and microbial activity, determining the salinity of the soil is a difficult task for both farmers and agricultural scientists. It has been demonstrated that inoculating plants with endophytic and PGP bacteria may decrease the detrimental effects of soil salinity (Kasim et al., 2016). These microorganisms aid growth of plant under saline conditions through several indirect and direct mechanisms. Furthermore, PGP bacteria’s biofilm has the ability to reduce the effects of salt stress. For example, compared to untreated controls, treating lettuce seedlings with *Azospirillum* enhanced both germination and vegetative growth in saline settings (Barassi et al., 2006). Similarly, applying *Pseudomonas stutzeri* to both salt-resistant and salt-sensitive chili peppers has been shown to lessen the harmful effects of salinity in the soil (Bacilio et al., 2016). While some microbial species can prevent biofilm formation on barley grains under saline stress, others do not have this ability (Kasim et al., 2016). Combining AM fungi with salt-tolerant bacteria can significantly enhance plants’ tolerance to salinity. For example, co-inoculating maize with *R. intraradices* and *Massilia* sp. RK4 improved root colonization and nutrient uptake under salt stress, highlighting the significant benefits of integrating fungal and microbial interactions to boost maize’s salinity tolerance (Krishnamoorthy et al., 2016).

#### 4.2.3 Manage maize temperature stress

To tackle the challenges in crop production due to climate change, innovative strategies such as using bacteria to manage heat and cold stress are essential. Temperature profoundly influences the microbe’s metabolism and physiology, with their enzymatic functions playing a crucial role in adapting to extreme conditions. These microorganisms employ sophisticated mechanisms to protect their membranes, nucleic acid and proteins. For example, they increase the production of heat-cold resistant enzymes and proteins in response to extreme temperatures. Molecular chaperones are particularly effective in safeguarding microorganisms from heat stress.

Microbes are classified based on their temperature preferences: psychrophilic bacteria, which thrive at or below 15°C, and psychrotrophic bacteria, which perform best at or above 15°C (Li et al., 2009). As a reaction to heat stress, microbes upregulate genes crucial for their survival. For instance, in *Alicyclobacillus acidoterrestris*, The DnaK gene is induced to express under heat stress, which produces heat shock proteins (HSPs) to shield the microorganism from thermal damage. This bacterium thrives in temperatures range from 23°C to 70°C, with optimal growth occurring between 45°C and 50°C. The production of HSPs is a key adaptation that helps it endure extreme temperatures.

Heat shock proteins are essential for surviving extreme heat stress. They help microbes endure heat stress by supporting proper nutrition, water intake, and enhancing photosynthesis. Trehalose, a sugar synthesized in response to thermal stress, provides protection against heat, cold, and oxidative damage. During heat stress, the levels of trehalose in bacteria and fungi increase significantly. Trehalose stabilizes proteins by preventing heat-induced denaturation and aggregation, thereby preserving their functional integrity. Additionally, trehalose is effective against freezing and dehydration, making it a valuable protective agent during drought conditions. Based on the particular plant and microbe, different metabolites are produced at different rates (Li et al., 2010).

#### 4.2.4 Heavy metal remediation in maize with the help of microbes

Microbes linked with plants, including rhizobacteria, mycorrhizae, firmicutes, and heavy metal-tolerant microorganisms, can significantly improve plant development and growth when exposed to metal stress. These microbes employ a range of strategies to manage metal stress, such as metal efflux, creating barriers to metal uptake, volatilization, sequestration through exopolysaccharides, enzymatic detoxification, and metal complexation. They also support plant health by lowering ethylene levels, produce growth regulators like ACC deaminase, IAA and controlling diseases (Glick, 2010). Additionally, they contribute to plant health through phosphate solubilization, siderophore production, nutrient mobilization and nitrogen fixation (Ahmad et al., 2011; Verma and Kuila, 2019).

Microbial biomass, both dead and living, can be used to remove heavy metals, with the characteristics and functional groups of bacterial and fungal cell walls being critical to this process (Vijayaraghavan and Yun, 2008), **Table 2**. Microbial bioaccumulation is an effective technique for extracting heavy metals from contaminated soils. certain microbial communities such as Actinobacteria, Firmicutes, Proteobacteria, have shown particular efficacy in removing higher concentrations of lead (Pb), arsenic (As) and manganese (Mn), from metal-polluted soils (Zhang et al., 2015). For instance, while elevated copper (Cu) levels adversely affected the growth of Vicia faba, the presence of rhizobia and PGPR mitigated these effects (Gould et al., 2018). Additionally, AM fungi can alleviate cadmium (Cd) stress by decreasing levels of malondialdehyde and hydrogen peroxide (Hashem et al., 2016).

**Table 2:** Microbial strains (Abiotic stress and PGP) for stress amelioration.

S. No.	Microbial strains	Crops	Response	References
**1.**	**Drought stress**			
	*Alcaligenes faecalis* AF3	*Zea mays*	Enhanced RW and protein	Naseem and Bano, 2014.
	*Azospirillum brasilense* SP-7 and *Herbaspirillum seropedicae* Z-152	*Zea mays*	Enhanced biomass, Nitrogen	Cohen et al., 2015.
	*Pseudomonas putida* H2-3	*Zea mays*	increased production of several hormones, such as EPS, which provide drought resistance.	Chieb and Gachomo, 2023.
	*Actinobacteria*	*Zea mays*	Illustrates drought induced alterations	Iqbal et al., 2023.
	*Piriformospora indica*	*Zea mays*	Increased root growth, canopy development, SPAD values.	Yun and Xu, 2018.
**2.**	**Salinity stress**			
	*Pseudomonas fluorescens*	*Zea mays*	Enhanced biomass, uptake of Na^+^ and K^+^ions	Nadeem et al., 2009.
	*Bacillus subtilis* (PM31)	*Zea mays*	promote growth, and alleviate salinity stress	Ali et al., 2023.
	*Pseudarthrobacter psychrotolerans* MHR1*, Pseudomonas simiae* MHR6, and *Acinetobacter calcoaceticus* MHR7	*Zea mays*	Growth of maize seedlings under simulated salt stress.	Liu et al., 2022.
**3.**	**Heavy metalstress**			
	*Pseudomonas fluorescens* 002	*Zea mays*	Alleviate Al stress	Qian et al., 2012.
	*Agrococcus terreus*	*Zea mays*	Potential for increasing growth in maize plants cultivated in soil contaminated with zinc and nickel.	Shahzad and Anam, 2023.
**4.**	**Temperature stress**			
	*Serratia nematodiphila, Paraburkholderia nodosa Burkholderia sensu stricto*	*Zea mays*	Plant growth-promoting effects.	Tang et al., 2020.

*Klebsiella* sp. and Enterobacter sp. is highly effective in tolerating metals and enhancing plant growth using producing growth-promoting chemicals while also removing cadmium, lead, and zinc from contaminated soils (Jing et al., 2014). Similarly, cadmium-resistant PGPB, likes and *Klebsiella* sp. BAM1, and *Micrococcus* sp. MU1 increase cadmium mobilization, root elongation, and overall plant growth in polluted environments (Prapagdee et al., 2013). From Pteris vittata Arsenic-resistant bacteria was isolated that solubilize phosphate further improve plant growth and nutrient uptake. Additionally, *Bradyrhizobium japonicum* E109 and *Azospirillum brasilense* Az39 rapidly colonize arsenic-contaminated soils, accumulate arsenic in them promote plant growth. And biomass. These examples highlight how PGPR can help plants thrive under heavy metal stress although lowering the concentration of heavy metals in plant tissues (Li et al., 2007; Armendariz et al., 2015).

### 4.3 Biotic stress

Maize output is greatly impacted by biotic stress, which is mostly caused by diseases and insect pests (Lodha et al., 2013). Aflatoxin contamination, sugarcane mosaic disease, ear rot, maize, northern leaf blight and rough dwarf disease are among the common illnesses that affect maize. Corn borers from Europe, the Mediterranean, and the tropics, along with maize weevil, which ruins stored grain, are the main pests that affect maize. In the US, maize anthracnose, the fungus *Colletotrichum graminicola* is the cause of these losses, which can reach $1 billion per year (Frey et al., 2011; Balmer et al., 2013). Furthermore, stalk-boring larvae of lepidopterans do enormous economic harm worldwide. Particularly troublesome is the European corn borer (ECB), which cause plant lodging during harvest by eating on kernels and digging into stalks. According to Tigar et al. (1994), maintenance Due to the extensive prevalence of the maize weevil, farmers in tropical and subtropical regions frequently experience grain damage of over 30% while storing their crops (Sitophilus zeamais Motsch).

#### 4.3.1 Northern leaf blight

Nearly every maize-growing location in the world is affected by northern leaf blight, a leaf disease caused by *Exserohilum turcicum*, a fungus. This disease is especially common in cooler climes with temperatures within 20° and 25°C, high relative humidity (90–100%), and low light levels (Wu et al., 2014). Yield losses have surpassed 50% in northern China, where *E. turcicum* infections are common (Ji et al., 2010). It has been proven that the fungicide systemic propiconazole successfully lessens the disease’s severity. Using tolerant cultivars like BH-540 and resistant hybrids like BH-660 can also aid in managing the illness. By lowering the amount of inoculum accessible for the following growing season, techniques including managing contaminated crop residues during the winter and in addition, switching out maize for non-host crops can help lower the disease load (Singh et al., 2014).

#### 4.3.2 Southern corn leaf blight

Southern corn leaf blight (SCLB), a serious foliar disease that affects maize, is brought on by the fungus *Cochliobolus heterostrophus* (Drechsler) Drechsler [synonym: *Helminthosporium maydis* Nisikado; anamorph: Bipolaris maydis (Nisikado) Shoemaker]. This is a common illness in hot, humid regions of the globe where maize is planted. There are three known races of the pathogen: The most prevalent race, regardless of the resistance genes or cytoplasm type, Race O infects all types of maize (Ullstrup, 1972). Race T, which is particularly virulent on Texas male-sterile cytoplasm (cms-T) cultivars, caused severe outbreaks in 1970 and 1971 (Ullstrup, 1972). China’s Races C is particularly harmful to cultivars of Charrua male-sterile cytoplasm (Wei et al., 1988). When compared to untreated plants, maize seedlings treated with *Azospirillum brasilense* show superior relative and absolute water content during drought circumstances. Water potential, proline, biomass, leaf area, and root growth accumulation in both leaves and roots are all enhanced by this treatment. In terms of protection, Bacillus cereus C1L was equivalent to Maneb (2 kg a.i/ha), a fungicide that is advised. Additionally, according to Fang et al. (2013), *Pseudomonas aurantiaca* shown strong antagonistic activity against *Bipolaris maydis*. Even when there were insufficient dithiocarbamate residues on the leaf surfaces to provide sufficient protection, *Bacillus cereus* CIL was successful in keeping southern leaf blight off of maize (Lai et al., 2016).

More than 20 distinct mold species have been linked to stalk and ear rot in maize, such as*, Trichothecium* spp. *F. verticillioides, Cladosporium* spp.*, Fusarium graminearum, Penicillium* spp., and *F. proliferatum.* According to van Egmond et al. (2007), these diseases are particularly common in places with high humidity and little rainfall, such the southern United States and certain lowland tropical regions. Mycotoxin-contaminated and moldy grains cause large yield losses and endanger the health of humans and livestock alike. Global proteomics was used by Mohammadi et al. (2011) to look at *F. graminearum* early infections in the sensitive line CO441 and The B73 line of inbred maize is tolerant.

Defence-related proteins, such as proteinase inhibitors, xylanase inhibitors, chitinases, a class III peroxidase, pathogenesis-related-10, and proteinase inhibitors, were found in high concentrations in infected creating kernels. The susceptible line’s kernel**s** had higher concentrations of these defence proteins, suggesting that these proteins play a vital defensive function against *F. graminearum*. Furthermore, root diseases brought on by *Macrophomina phaseolina Fusarium moniliforme*, and *F. graminearum*, have been demonstrated to be made easier by the creation of antibiotics and siderophores by strains such as *Pseudomonas fluorescens* spp. M23 + *Bacillus* sp. MRF and *Bacillus* sp. MR-11(2) + *Bacillus* sp. MRF (Pal et al., 2001).

Researchers examined resistant and susceptible inbred lines of maize in order to understand the gene and metabolite responses to *Fusarium* infection (Campos-Bermudez et al., 2013). Following the *Fusarium inoculation*, microarray examination of maize kernels showed that the resistant line showed only slight alterations in metabolite levels or gene expression. On the other hand, in response to the infection, the susceptible lines displayed notable changes in defence-related gene expression. These findings suggest that innate defensive mechanisms that ward off fungal infection account for a significant portion of maize resistance (Campos-Bermudez et al., 2013). Subsequent examination of these maize genotypes showed that prior to infection, the susceptible line had lower levels of gene expression linked to defence, while the resistant line had higher levels.

According to this, susceptible lines must upregulate these defences as a reaction to infection, whereas resistant maize lines maintain elevated amounts of genes and proteins linked to defence both before and during infection (Alessandra et al., 2010). Furthermore, modern methods like stereo fluorescence microscopy and PEG-CaCl2 mediated transformation are becoming more and more important for researching stalk and disease associated with ear rot in maize (Mesterhazy et al., 2020).

#### 4.3.3 Stalk rot of pythium

Throughout the lowlands of southern Nepal and northern India, stalk rot poses a serious threat and seriously damages crops. The development of the disease is strongly impacted by host variables as well as environmental factors. At temperatures between 30 and 35 degrees Celsius and relative humidity levels between 80 and 100%, the pathogen grows and most effectively causes disease. Furthermore, areas that are wet, low-lying, or poorly drained accelerate the rate of disease. The danger infection is increased by high plant densities (≥ 60,000 plants per hectare) and plant age, especially in the pre-flowering period (Diwakar et al., 1980). In order to effectively manage stalk rot, it is necessary to maintain sufficient soil nitrogen levels and mitigate plant stress through appropriate soil drainage. Resistance to pyrethium stalk rot in the Qi319 maize inbred line has been related to two independently inherited dominant genes (Kenganal et al., 2017).

#### 4.3.4 Bacterial stalk rot

In India, bacterial stalk rot has grown to be a serious problem for maize crops produced in the kharif season (Kumar, 2015). This ailment is worsened by the kharif season, which falls during the monsoon season every year. Though Prasad first identified *E. dissolvens* as the origin of the sickness in 1930, the disease’s symptoms are more akin to those of *E. chrysanthemi pv. zeae*. It became well-known in 1969 amid a serious epidemic in the Mandi area of Himachal Pradesh. The disease is spread by precipitation and runoff, and reports of cases have been made in many parts of the world (Prasad, 1930; Martinez-Cisneros et al., 2014). Based on Prasad (1930) and Samson et al. (2005), there are three bacterial pathogens that cause maize stalk rot: *Pseudomonas syringae pv. Lapsa, E. dissolvens, Pseudomonas syringae, E. chrysanthemi pv. zeae, E. dissolvens*, and *Pseudomonas syringae pv. lapsa.*, which has a broad host range and causes soft rot, poses significant management challenges (Bradbury, 1986). Severe infections can cause maize plants to collapse and emit a foul odor, resulting to yield losses of between 21% to 98% (Thind and Payak, 1978). Although complete resistance to these pathogens has not yet been achieved, researchers are focusing on identifying quantitative trait loci linked with resistance (Canama and Hautea, 2010). Control strategies include applying chemical treatments, employing biological control methods, and developing host plant resistance. For chemical control, chlorinating irrigation water or soil drenching with bleaching powder before flowering is recommended, and copper oxychloride formulations have also been effective. In biological control, *Pseudomonas fluorescens* has shown potential against *Erwinia chrysanthemi* in laboratory conditions, though its effectiveness in field settings is still limited (Singh et al., 2020).

#### 4.3.5 Head smut

An uncommon maize disease seen in Nepal is head smut, which is brought on by *Sporisorium reiliana* (*Kuhn*) Langdon Full (previously known as *Sphacelotheca reiliana* (*Kuhn*) Clinton or *Ustilago reiliana Kuhn*). In 1966, the illness was initially reported in Ilam (Khadka and Shah, 1967). It’s common in Nepal’s hilly areas and is typified by a characteristic black sori, which is frequently accompanied by phyllody or other irregularities in the ears and tassels (Gurung et al., 1985). Usually, smut balls or leafy growths replace the tassels or ears completely or in part. During emergence or the seedling stage, soil-borne spores infect maize plants, which subsequently spread systemically through the meristem of the plant (Xu et al., 1999). Farmers have reported difficulties with smut in their fields, prompting the implementation of a several different management techniques, including crop rotation, seed treatments, foliar fungicide applications, fertility adjustments, sanitation and biological controls. However, host resistance continues to be the most successful strategy for managing common smut, particularly in areas where *U. maydis* is widespread. Symptoms are first noticeable on the tassels, but even plants with seemingly normal tassels can be infected, with smutted ears only becoming visible at harvest.

#### 4.3.6 Common rust

The rust disease that *Puccinia sorghi* causes in maize is prevalent in certain areas including South China, Nepal, Bhutan, and northern India. In Nepal, infections on *Oxalis* species are rare, whereas in India, *Oxalis corniculata* has shown susceptibility to artificial inoculation with *P. sorghi* teliospores, though natural infections have not been observed. Significant rust outbreaks have been noted in Bihar, India, since the early 1970s, especially affecting winter plantings of susceptible hybrids such as Ganga Safed-2 and Hi-starch (Sharma et al., 1993). The disease development is particularly higher in summer maize within mountainous regions and valleys, with fewer cases in the Terai plains; however, it has also affected winter and spring maize in Nepal’s Terai region (Kushalappa and Hegde, 1970), typically appearing either when tasseling or at the knee-high stage. In Nepal, local varieties of maize are extremely vulnerable, suggesting that common rust existed before its formal documentation in 1964 (Manandhar, 1972). Rust-induced yield losses can range from 6% to 32% (Sharma et al., 1982). Research by Sharma and Payak (1979) has indicated that rust resistance is polygenic, involving multiple genes that contribute to resistance. To manage maize rustfoliar fungicide spraying and the adoption of resistant hybrids are standard practices. Additionally, cultural methods can be effective, particularly in areas where rust spores overwinter on plant debris or diseased Oxalis species (Utpal and Ritika, 2015).

#### 4.3.7 Downy mildews

The downy mildew (DM) species that are most common in the region are *P. sorghi, Java DM, P. maydis*, Brown stripe DM (*Sclerophthora rayssiae* var. *zeae*), *Sorghum DM, P. sacchari*, and *Peronosclerospora philippinensis*. These pathogens show a significant threat to the production of maize in South and Southeast Asia. The disease is endemic in the warm, humid Terai region (Shah, 1976). Typically, infection rates range from 10% to 20%, but can escalate to 30% to 60% during periods of excessive moisture and humidity (Manandhar, 1972). Downy mildew usually manifests when plants are 3 to 4 weeks old, and severe infections can cause a 10% to 20% reduction in grain yield. Most severe outbreaks occur in crops sown late in the season (June-July) (Manandhar, 1975). Since 1975, signs of crazy top, such as deformed plants, have been seen in the inner Terai, although the precise pathogen is still unknown. Agents like *Trichoderma harzianum, Bacillus subtilis, Gliocladium virens*, and *Trichoderma viride* are used for biological control. Spore germination can be inhibited more successfully in dual cultures of *T. viride* with *T. harzianum* or *B. subtilis* than in single cultures. Out of all of these, *T. viride* combined with *B. subtilis, T. harzianum* works best to reduce downy mildew infection, whereas *G. virens* works less well. Chemically, the fungicide Apron has been found to be the most effective treatment overall (Alzahrani et al., 2021).

#### 4.3.8 Philippine downy mildew

The plains of Nepal, the Philippines, Laos, northern Vietnam, and northern India are among the places where this disease is most common. Within India, it was initially recorded in 1912. According to Exconde (1970), this particular strain of DM is quite aggressive, frequently resulting in yield decreases of 40% to 60% and disease incidence rates of 80% to 100%. In Nepal, the disease reached epidemic levels in 1987, causing up to 50% yield losses, with late-sown crops suffering the most severe damage (Shah and Tuladhar, 1971). The infection commonly originates from the wild grass *Saccharum spontaneum*, which can be found growing naturally or as a planted barrier around fields. Managing this grass can help control DM. Chemical control strategies include using protectants, applying radiant sprays to leaves, treating soil, and treating seeds to manage and eliminate *P. philippinensis* (Fornah et al., 2020).

#### 4.3.9 Sorghum downy mildew

First discovered in 1905 in Pune in teosinte (*Zea mays* spp. *mexicana*), the pathogen was later discovered in sorghum in 1907 in India (Butler, 1907; Uppal and Desai, 1932). Since the 1960s, it has been reported causing damage to *Zea mays* and *Sorghum bicolor* globally. *P. sorghi* comprises three distinct strains: one that affects maize, one that targets sorghum, and one that affects both crops. India has observed all three variants, while only the strain specific to maize has been identified in Thailand (Payak et al., 1979). Only four of the fifteen alleles shared between the Thai sample and other *P. sorghi* isolates, according to isozyme analysis conducted by Bonde et al. (1984), indicates that *P. sacchari* and *P. philippinensis* is the complex to which the Thai isolate is more closely linked than *P. sorghi*. When resistant types are lacking, the disease can cause significant harm. Asia has embraced the downy mildew-resistant cultivar Suwan 1, which was created in Thailand in 1973 and is still very successful. Suwan 1 is available in the following countries: Philippines, Vietnam, South China, India, South China, Indonesia, Laos, Nepal, Burma, and the Bhutan. Biological control techniques employing antagonistic bacteria and the application of metalaxyl fungicides to seeds are examples of management strategies. *B2, Brevibacillus brevis 57*, and *Pseudomonas fluorescens* Pf1*, Bacillus subtilis G1, and Bacillus amyloliquefaciens* are examples of effective biocontrol agents (Sadoma et al., 2011).

#### 4.3.10 Brown stripe downy mildew

Following its discovery in India (Subedi, 2015), reports of the illness have been also made in Thailand, Pakistan, Burma, and Nepal (Frederiksen and Renfro, 1977). Other than South and Southeast Asia, no records exist for it. The disease mainly affects areas in the northern Indian Himalayan region that are less than 1500 meters above sea level. It can result in yield losses of as much as 63% in Uttar Pradesh’s Tarai region (Lim et al., 2023). Teosinte, *Digitaria sanguinalis* in India, and *D. bicornis* in Thailand have also been shown to harbor the disease, in addition to maize. While separate chemical and biological control techniques have shown to be ineffective, an integrated management approach that incorporates preventive measures and chemical and biological treatments is essential for managing the Brown Stripe Downy Mildew (Lal et al., 1980).

#### 4.3.11 Brown spot

*Physoderma maydis*is the cause of brown spot disease, is a widespread affliction that commonly appears around the tasseling stage of maize. It impacts several parts of the plant, such as stalks, leaf sheaths, leaves, and occasionally, the outer husks. This disease thrives in regions with high average temperatures and substantial rainfall. Early signs of infection include small, chlorotic patches that form distinct bands of affected and healthy tissue on the leaf blades. Initial leaf lesions present as chlorotic dots, while spots on the midribs are typically round and dark brown. In severe cases, brown lesions can develop on nodes and internodes, sometimes merging to cause stalk rot and plant lodging (CIMMYT Maize Program, 2004). Fungicides including propiconazole, trifloxystrobin, azoxystrobin, iprodione, and carbendazim are applied as part of management techniques. Furthermore, prior to planting, seeds should be treated with hot water at 53–54°C for 10–12 minutes to help prevent the primary infection during the development of the seedlings.

#### 4.3.12 Rough dwarf disease of maize

A severe ailment known as maize rough dwarf disease (MRDD) significantly lowers maize output. Three distinct pathogens cause this disease: Maizerough dwarf virus (MRDV), rice black-streaked, Mal de Ro Cuarto virus (MRCV), and dwarf virus (RBSDV) (Dovas et al., 2004). Whereas MRCV is the main culprit in South America, MRDV is the principal pathogen causing MRDD in Europe. The most common cause of MRDD in China is RBSDV, wherein *Laodelphax striatellus* spreads throughout (Zhang et al., 2021). Even though MRDD is a serious disorder, not much study has been done using omics technology. Using 2-DE and MS/MS, a comparative proteome study was conducted to examine the differences between virus-infected and healthy maize leaves. This study found that pathways related to glycolysis, starch metabolism, and morphogenesis were notably upregulated in maize infected with RBSDV compared to healthy plants (Li et al., 2011).

#### 4.3.13 Sugarcane mosaic disease

Virus-induced diseases can significantly impact food production, with the Sugarcane mosaic virus (SCMV) being a major contributor to yield losses in both grain and forage crops. SCMV has posed particular challenges for maize cultivation in Argentina (Perera et al., 2009) and China (Xu et al., 2008). A DIGE-based proteomics method was used to examine the protein profiles of types of maize that are both SCMV-resistant and -susceptible in order to investigate the effects of SCMV infection on maize (Wu et al., 2013). Ninety-three proteins were found to have changed expression as a result of infection; Numerous proteins in question play roles in energy metabolism, photosynthesis, and stress responses, and carbon fixation. The majority of the SCMV-responsive proteins in the maize cultivars Siyi and Mo17 were found to be located in the cytoplasm, chloroplast membranes, 2-DE and MALDI-TOF-MS/MS studies (Wu et al., 2013). Additional investigation of these proteins may provide more comprehensive understanding of the connections between SCMV and maize.

#### 4.3.14 Nematodes

Nematode species damage maize; in some maize-growing locations, particularly troublesome is the cyst nematode *Heterodera zeae Koshy*, Swarup, and Sethi (Cui et al., 2024). Many strategies are used to control nematode infestations: using pesticides, rotating crops, adding fertilizers, solarizing the soil, and planting resistant types. Soil solarization has shown to be particularly successful among these techniques in managing a variety of worms and illnesses spread via the soil (Mandal et al., 2021).

#### 4.3.15 Contamination with aflatoxin

Maize crops are seriously threatened by aflatoxin, a carcinogenic material that is mostly produced by the fungus *Aspergillus flavus* (Klich, 2007). This fungus grows best in warm, humid circumstances, which worsen ear rot and increase the generation of aflatoxin. Hot, dry weather also increases contamination. Aflatoxin contamination induced by *A. flavus* is a frequent problem that causes large losses in maize production worldwide. Abiotic factors that exacerbate aflatoxin contamination include heat and drought. Aflatoxin levels might be lowered and *A. flavus* infection could be mitigated by increasing host plant resistance. In a proteome analysis, rachises from susceptible and resistant genotypes of maize showed increased amounts of proteins linked to the metabolism of phenylpropanoid and abiotic stress in the resistant line. In contrast, the susceptible line had elevated levels of pathogenesis-related proteins, indicating that resistant maize employs constitutive defences, whereas susceptible maize relies on inducible defences. Distinct differences in stress and defence protein expression were observed between 10- and 35-days post-infection (Pechanova et al., 2011).

##### 4.3.15.1 Plant growth-promoting rhizobacteria

Plant growth-promoting rhizobacteria (PGPRs) play a critical role in augmenting plant health and growth via diverse direct and indirect mechanisms. By improving soil conditions and generating growth-promoting compounds that increase the accessibility of vital minerals such as potassium, calcium, and phosphorus PGPRs directly increase fertility of soil (Tabassum et al., 2017; Naik et al., 2019). Additionally, they provide plant growth regulators, which help plants develop.

PGPRs also encourage plant growth in a number of indirect methods. They also create phytohormones that enable biological nitrogen fixation, increase phosphate and potassium availability, and produce gibberellins, ethylene, abscisic acid, cytokinins, and auxins. PGPRs help regulate or remove infections by creating biological control agents, which enhances the growth environment. Additional indirect mechanisms that break down fungal cell walls include lytic enzymes like chitinases and glucanases, competition for resources, and antibiosis (Bhattacharyya, 2012).

**Figure 4:**
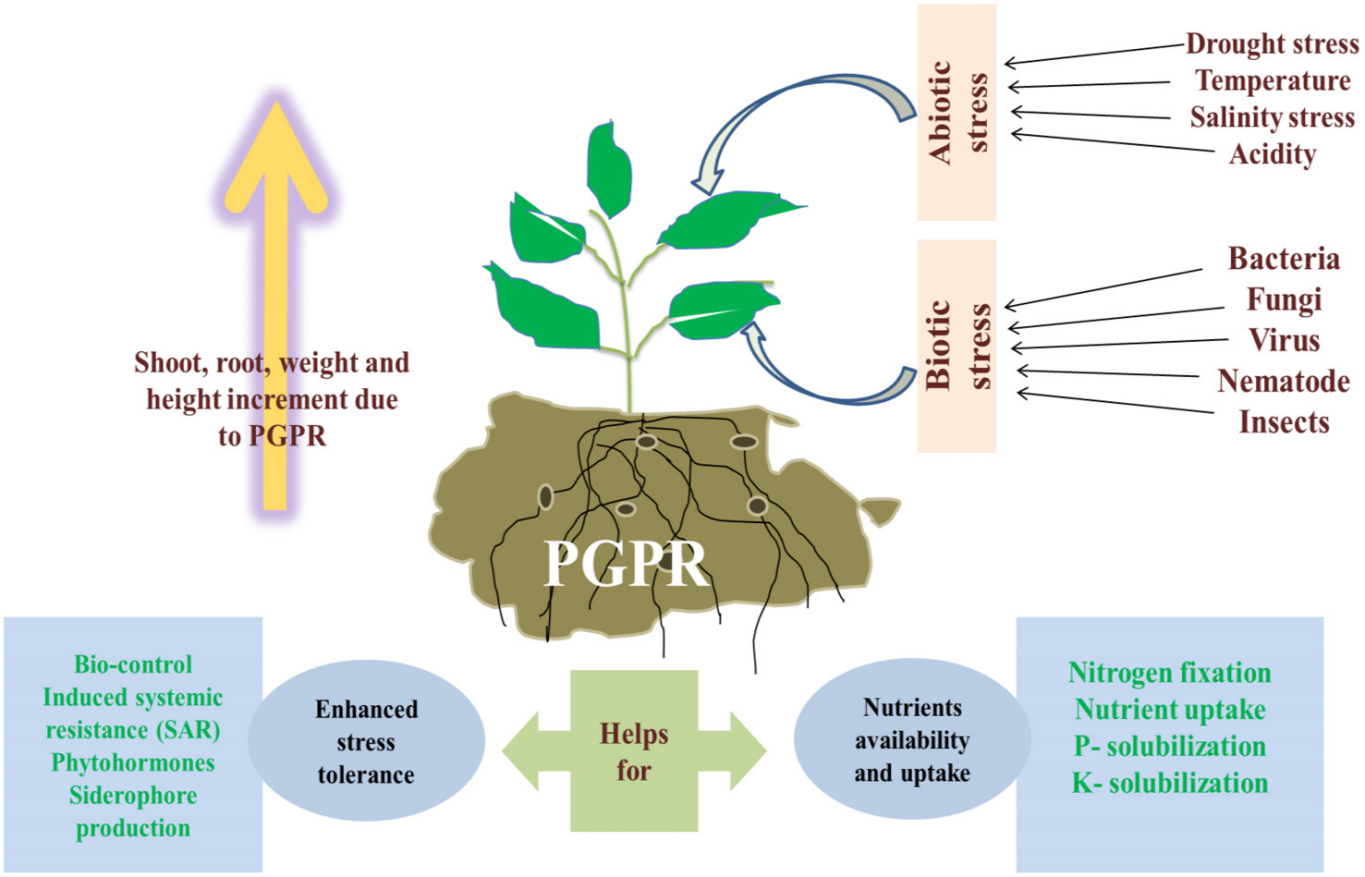
Plant interactions in the rhizosphere and PGPR.

By lowering the chance of infections and strengthening the plant’s natural defenses against pathogens, PGPRs can further encourage plant growth (Tabassum et al., 2017). For example, mechanisms like antibiotic-induced systemic resistance (ISR) and the production of siderophores contribute to these indirect benefits, as illustrated in **Figure 4**. These mechanisms can lead to systemic resistance across the plant or localized antagonistic effects against soil-borne diseases. Antagonistic rhizobacteria generate substances such as antibiotics and siderophores, which aid in disease management and indirect growth enhancement. ISR, akin to pathogen induced boosts the resilience of uninfected plant tissues, systemic acquired resistance (SAR), Rhizobacteria induce resistance through plant responses to ethylene and jasmonic acid or through the salicylic acid dependent SAR pathway. Genera like Pseudomonas and Bacillus are recognized for their ability to induce ISR and exhibit antagonistic properties. These beneficial rhizobacteria, which enhance plant resistance and act as antagonists, can be employed to create innovative inoculants that integrate multiple mechanisms, thus enhancing the effectiveness of biocontrol methods in agricultural systems.

An increase in fertilizer use is predicted as maize yield rises, which will drive up production prices and cause environmental problems. PGPR, on the other hand, have a history of success in increasing crop yields and growth. For instance, it has been demonstrated that *Bacillus safensis, Brevundimonas vesicularis, Bacillus pumilus*, and *Paenibacillus alvei*, increase yields between 24 to 34% (Breedt et al., 2017). *Bradyrhizobium japonicum* and *Azospirillum brasilense* together enhanced seed germination and early plant growth, as shown by Cassán et al. (2009). Kuan et al. (2016) discovered that bacteria that promote plant development could function as a biological substitute for atmospheric nitrogen fixation, leading to a possible 30.9 percent increase in agricultural yields and a decrease in the requirement for nitrogen fertilizers. Additionally, PGPR inoculants have been shown by Di Salvo et al. (2018) to improve grain yield and growth in cereal crops like maize. Despite these advantages, there is still much to learn regarding the ways in which microorganisms and plants interact, which emphasizes the need for more study on microbial ecology in the rhizosphere of various agricultural methods. Furthermore, whereas certain PGPR are known to generate IAA and enhance shoot and root weight as well nutrient intake to promote maize growth, other activities, such phosphorus solubilization, are still poorly understood (Lobo et al., 2019).

##### 4.3.15.2 Nano-based approach

The integration of nanotechnology in agriculture is growing, especially with the introduction of nanofertilizers (NFs) (Das et al., 2024). According to Liu and Lal (2015), these consist of substances such as Si- and Ti-based nanoparticles (NPs) and valuable metals like gold (Au-NPs). Particles between 1 and 100 nm in size must be classified as NFs because of their unique mobility, reactivity, and possible toxicity compared to bulk materials (Šebesta et al., 2022). Although their precise mechanisms are still somewhat unknown, some types of NFs, such as the non-nutrient stimulant TiO2-NPs and the micronutrient ZnO-NPs, have demonstrated the ability to improve crop growth and yield (Kolenčík et al., 2021).

Because NFs have a balanced agronomic impact and require less application rates and more progressive nitrogen release, they are beneficial for the environment. Studies reveal that applying NPs, particularly ZnO-NPs, and TiO2-NPs, topically can improve photosynthesis and yield in plants like foxtail millet, spinach, and sunflower. Nevertheless, little is known about their effects on the nutritional quality of humans and the possible health risks posed by NP residues building up in edible plant parts (Holišová et al., 2019; Ernst et al., 2024). Grains of interest for NP uses include maize, which is the third most eaten grain worldwide. Rich in nutrients, maize contains minerals that are critical to human health, such as potassium, calcium, zinc, and iron. Certain minerals, such as phytic acid, which contains phosphorus, can prevent the absorption of nutrients. Si and Ti are examples of NFs that have been connected to enhanced plant development, stress resistance, and antifungal qualities; nevertheless, because of their potential toxicity, other NPs, such as Au-NPs, may be hazardous (Ernst et al., 2024).

## 5 Conclusion

As the world’s population keeps increasing, agriculture remains crucial into meeting various needs. Historically, crops were grown using natural methods without pesticides, but yields were inadequate to support the rising population. As a result, farmers turned to chemical fertilizers, which improved yields but disrupted natural microflora and ecosystems. To address these issues, scientists are now focusing on bio-fertilizers for large-scale crop enhancement. Research indicates that bio-fertilizers are more effective than chemical fertilizers in boosting crop yields. Utilizing a consortium of natural bacterial cultures proves to be more beneficial than using single strains, as these microbial communities enhance productivity through their metabolic activities. Improving crop predictability is another benefit of these biological approaches. To date, 251 beneficial microorganism genes have been successfully transferred to crops, offering both biotic and abiotic resistance. The development of new microbial consortia is expected to further enhance crop productivity through advances in microbial biotechnology. Clear understanding of communication within consortium inoculants will improve their effectiveness in acclimatizing within the rhizosphere. Maize plants irrigated with industrial and municipal waste host heavy metal-resistant bacteria in their rhizosphere and endorhiza, which provide several benefits, including promoting plant growth. These heavy metal- and salinity-resistant PGP (plant growth-promoting) bacteria can help lower the concentrations of heavy metals in treated plants and lessen the stress that wastewater causes to plants. To assess these strains’ efficacy in contaminated soils in both greenhouse and field settings, more investigation is required. It is advised that PGPR be used more widely in agriculture due to its benefits in bio-fertilization, bio-control, and bioremediation—all of which increase crop output and ecosystem health. As technology advances and research develops, PGPR usage is expected to become more widespread, contributing to stable and productive agroecosystems and advancing agricultural practices.

## References

[B1] Adams T. R. Chambers S. A. Daines R. J. Gordon-Kamm W. J. Kaush A. P. Lemaux P. G.. (1999). Methods and composition for the production of stably transformed fertile monocot plants and cells thereof. US Patent 5874265.

[B2] Adebayo M. A. Menkir A. Gedil M. Blay E. Gracen V. Danquah E.. (2015). Diversity assessment of drought tolerant exotic and adapted maize (*Zea mays* L.) inbred lines with microsatellite markers. J. Crop Sci. Biotechnol. 18, 147–154. doi: 10.1007/s12892-014-0076-3

[B3] Agunbiade V. F. Babalola O. O. (2023). Endophytic and rhizobacteria functionalities in alleviating drought stress in maize plants. Plant Prot. Sci. 59, 1–18. doi: 10.17221/61/2022-PPS

[B4] Ahmad A. Ghufran R. Zularisam A. W. (2011). Phytosequestration of metals in selected plants growing on a contaminated Okhla industrial areas, Okhla, New Delhi, India. Water Air Soil pollut. 217, 255–266. doi: 10.1007/s11270-010-0584-9

[B5] Alam P. Balawi T. A. Faizan M. (2022). Salicylic acid’s impact on growth, photosynthesis, and antioxidant enzyme activity of triticum aestivum when exposed to salt. Molecules 28, 100. doi: 10.3390/molecules28010100 36615299 PMC9821804

[B6] Alessandra L. Luca P. Adriano M. (2010). Differential gene expression in kernels and silks of maize lines with contrasting levels of ear rot resistance after *Fusarium verticillioides* infection. J. Plant Physiol. 167, 1398–1391. doi: 10.1016/j.jplph.2010.05.015 20650545

[B7] Ali B. Hafeez A. Afridi M. S. Javed M. A. (2023). Bacterial-Mediated Salinity Stress Tolerance in Maize (*Zea mays* L.): A Fortunate Way toward Sustainable Agriculture. Omega 8, 23. doi: 10.1021/acsomega.3c00723 PMC10275368 37332827

[B8] Alotaibi M. (2023). Climate change, its impact on crop production, challenges, and possible solutions. Notulae Botanicae Horti Agrobotanici Cluj-Napoca 51, 13020. doi: 10.15835/nbha51113020

[B9] Alzahrani M. A. Sadoma H. H. M. Mathew S. Alghamdi S. Malik J. A. Anwar S. (2021). “Retrospective analysis of antimicrobial susceptibility of uropathogens isolated from pediatric patients in tertiary hospital at al-baha region, saudi arabia,” in Healthcare, vol. 9, 1564. doi: 10.3390/healthcare9111564 34828610 PMC8623104

[B10] Armendariz A. L. Talano M. A. Oller A. W. Medina M. I. Agostini E. (2015). Effect of arsenic on tolerance mechanisms of two plant growth-promoting bacteria used as biological inoculants. J. Environ. Sci. 33, 203–210. doi: 10.1016/j.jes.2014.12.024 26141894

[B11] Atkinson N. J. Urwin P. E. (2012). The interaction of plant biotic and abiotic stresses: from genes to the field. J. Exp. Bot. 63, 3523–3543. doi: 10.1093/jxb/ers100 22467407

[B12] Bacilio M. Moreno M. Bashan Y. (2016). Mitigation of negative effects of progressive soil salinity gradients by application of humic acids and inoculation with *Pseudomonas stutzeri* in a salt-tolerant and a salt-susceptible pepper. Appl. Soil Ecol. 107, 394–404. doi: 10.1016/j.apsoil.2016.04.012

[B13] Badu-Apraku B. Fakorede M. B. Talabi M. O. Akaogu R. O. Akaogu I. C. Akinwale R. O.. (2016). Gene action and heterotic groups of early white quality protein maize inbreeds under multiple stress environments. Crop Sci. 56, 183–199. doi: 10.2135/cropsci2015.05.0276

[B14] Badu-Apraku B. Talabi A. O. Ifie B. E. Chabi Y. C. Obeng-Antwi K. Haruna A.. (2018). Gains in Grain Yield of Extra-Early Maize during Three Breeding Periods under Drought and Rainfed Conditions. Crop Sci. 58, 2399–2412. doi: 10.2135/cropsci2018.03.0168 33343015 PMC7680940

[B15] Bal H. B. Nayak L. Das S. Adhya T. K. (2013). Isolation of ACC deaminase producing PGPR from rice rhizosphere and evaluating their plant growth promoting activity under salt stress. Plant Soil 366, 93–105. doi: 10.1007/s11104-012-1402-5

[B16] Balmer D. Flors V. Glauser G. Mauch-Mani B. (2013). Metabolomics of cereals under biotic stress: current knowledge and techniques. Front. Plant Sci. 4, 82. doi: 10.3389/fpls.2013.00082 23630531 PMC3632780

[B17] Barassi C. A. Ayrault G. Creus C. M. Sueldo R. J. Sobrero M. T. (2006). Seed inoculation with *Azospirillum mitigates* NaCl effects on lettuce. Scientia Hortic. 109, 8–14. doi: 10.1016/j.scienta.2006.02.025

[B18] Barea J. M. (2015). Future challenges and perspectives for applying microbial biotechnology in sustainable agriculture based on a better understanding of plant-microbiome interactions. J. Soil Sci. Plant Nutr. 15, 261–282. doi: 10.4067/S0718-95162015005000021

[B19] Barman M. Paul S. Choudhury A. G. Roy P. Sen J. (2017). Biofertilizer as prospective input for sustainable agriculture in India. Int. J. Curr. Microbiol. Appl. Sci. 6, 1177–1186. doi: 10.20546/ijcmas

[B20] Basu S. Ramegowda V. Kumar A. Pereira A. (2016). Plant adaptation to drought stress. F1000Research 5. doi: 10.12688/f1000research PMC4937719 27441087

[B21] Berta G. Copetta A. Gamalero E. Bona E. Cesaro P. Scarafoni A.. (2014). Maize development and grain quality are differentially affected by mycorrhizal fungi and a growth-promoting pseudomonad in the field. Mycorrhiza 24, 161–170. doi: 10.1007/s00572-013-0523-x 23995918

[B22] Bhattacharyya J. (2012). Plant growth-promoting rhizobacteria (PGPR): Emergence in agriculture. World J. Microbiol. Biotechnol. 28, 1327–1350. doi: 10.1007/s11274-011-0979-9 22805914

[B23] Bhusal B. Poudel M. R. Rishav P. Regmi R. Neupane P. Bhattarai K.. (2021). A review on abiotic stress resistance in maize (*Zea mays* L.): effects, resistance mechanisms and management. J. Biol. Today’s World 10, 1–3.

[B24] Bonde M. R. Peterson G. L. Dowler W. M. May B. (1984). Isozyme analysis to differentiate species of *Peronosclerospora* causing downy mildews of maize. Phytopathology 74, 1278–1283. doi: 10.1094/Phyto-74-1278

[B25] Bradbury J. F. (1986). Guide to plant pathogenic bacteria (Farnham Royal, Slough: CAB international).

[B26] Breedt G. Labuschagne N. Coutinho T. A. (2017). Seed treatment with selected plant growth-promoting rhizobacteria increases maize yield in the field. Ann. Appl. Biol. 171, 229–236. doi: 10.1111/aab.12366

[B27] Bromham L. Saslis-Lagoudakis C. H. Bennett T. H. Flowers T. J. (2013). Soil alkalinity and salt tolerance: adapting to multiple stresses. Biol. Lett. 9, 20130642. doi: 10.1098/rsbl.2013.0642 23925838 PMC3971713

[B28] Brookes G. Barfoot P. (2010). Global impact of biotech crops: Environmental effects 1996-2008. AgBioForum 13 (1), 76–94. doi: 10.4161/gmcr.20061

[B29] Bui E. N. (2013). Soil salinity: a neglected factor in plant ecology and biogeography. J. arid environ. 92, 14–25. doi: 10.1016/j.jaridenv.2012.12.014

[B30] Burlakoti S. Devkota A. R. Poudyal S. Kaundal A. (2024). Beneficial plant–microbe interactions and stress tolerance in maize. Appl. Microbiol. 4, 1000–1015. doi: 10.3390/applmicrobiol4030068

[B31] Butler E. J. (1907). Some diseases of cereals caused by Sclerospora graminicola (Calcutta: Thacker, Spink Company).

[B32] Cai F. Zhang Y. Mi N. Ming H. Zhang S. Zhang H.. (2020). Maize (Zea mays L.) physiological responses to drought and rewatering, and the associations with water stress degree. Agric. Water Manage. 241, 106379. doi: 10.1016/j.agwat.2020.106379

[B33] Campos-Bermudez V. A. Fauguel C. M. Tronconi M. A. Casati P. Presello D. A. Andreo C. S. (2013). Transcriptional and metabolic changes associated to the infection by *Fusarium verticillioides* in maize inbreds with contrasting ear rot resistance. PloS One 8, e61580. doi: 10.1371/journal.pone.0061580 23637860 PMC3630110

[B34] Canama A. O. Hautea D. M. (2010). Molecular mapping of resistance to bacterial stalk rot (*Pectobacterium chrysanthemi* pv. zeae Burk., McFad. and Dim.) in tropical white maize (*Zea mays* L.). Philippine Agric. Scientist 93, 429–438.

[B35] Canton H. (2021). Food and agriculture organization of the united nations–FAO. Europa directory International organizations (Routledge) 2021, 297–305.

[B36] Casati P. Campi M. Morrow D. J. Fernandes J. F. Walbot V. (2011a). Transcriptomic, proteomic and metabolomic analysis of UV-B signaling in maize. BMC Genomics 12, 1–17. doi: 10.1186/1471-2164-12-321 PMC3141669 21679461

[B37] Casanovas E. M. Barassi C. A. Sueldo R. J. (2002). Azospiriflum inoculation mitigates water stress effects in maize seedlings. Cereal Res. Commun. 30 (3), 343–350. doi: 10.1007/BF03543428

[B38] Casati P. Morrow D. J. Fernandes J. Walbot V. (2011b). UV-B signaling in maize: transcriptomic and metabolomic studies at different irradiation times. Plant Signaling Behav. 6, 1926–1931. doi: 10.4161/psb.6.12.18164 PMC3337180 22105027

[B39] Cassán F. Perrig D. Sgroy V. Masciarelli O. Penna C. Luna V. (2009). *Azospirillum brasilense* Az39 and Bradyrhizobium japonicum E109. European Journal of Soil Biology 45, 28. doi: 10.1016/j.ejsobi.2008.08.005

[B40] Castaldini M. Turrini A. Sbrana C. Benedetti A. Marchionni M. Mocali S.. (2005). Impact of Bt corn on rhizospheric and soil eubacterial communities and on beneficial mycorrhizal symbiosis in experimental microcosms. Appl. Environ. Microbiol. 71, 6719–6729. doi: 10.1128/AEM.71.11.6719-6729.2005 16269702 PMC1287690

[B41] Chieb M. Gachomo E. G. (2023). The role of plant growth promoting rhizobacteria in plant drought stress responses. BMC Plant Biol. 23, 407. doi: 10.1186/s12870-023-04403-8 37626328 PMC10464363

[B42] Cohen A. C. Bottini R. Pontin M. Berli F. J. Moreno D. Boccanlandro H.. (2015). *Azospirillum brasilense* ameliorates the response of *Arabidopsis thaliana* to drought mainly via enhancement of ABA levels. Physiol. Plant. 153, 79–90. doi: 10.1111/ppl.12221 24796562

[B43] Cohen O. Drake J. J. Kashyap V. L. Saar S. H. Sokolov I. V. Manchester W. B.. (2009). Interactions of the magnetospheres of stars and close-in giant planets. Astrophysical J. 704 (2), L85. doi: 10.1088/0004-637X/704/2/L85

[B44] Cui J. Ren H. Wang B. Chang F. Zhang X. Meng H.. (2024). Hatching and development of maize cyst nematode Heterodera zeae infecting different plant hosts. J. Integr. Agric. 23, 1593–1603. doi: 10.1016/j.jia.2023.04.042

[B45] Dartora J. Guimarães V. F. Menezes C. R. Freiberger M. B. Castoldi G. Gonçalves E. (2016). Maize response to inoculation with strains of plant growth-promoting bacteria. Rev. Bras. Engenharia Agrícola e Ambiental 20, 606–611. doi: 10.1590/1807-1929/agriambi.v20n7p606-611

[B46] Das R. Kumar P. Agrawal S. Singh K. Singh N. Singh S.. (2024). Nanoparticles for crop improvement and management. Sustainable Agriculture, De Gruyter, pp. 69–84. doi: 10.1515/9783111234694-005

[B47] Dhawi F. Datta R. Ramakrishna W. (2015). Mycorrhiza and PGPB modulate maize biomass, nutrient uptake and metabolic pathways in maize grown in mining-impacted soil. Plant Physiol. Biochem. 97, 390–399. doi: 10.1016/j.plaphy.2015.10.028 26546782

[B48] Di Salvo L. P. Cellucci G. C. Carlino M. E. de Salamone I. E. G. (2018). Plant growth-promoting rhizobacteria inoculation and nitrogen fertilization increase maize (*Zea mays* L.) grain yield and modified rhizosphere microbial communities. Appl. Soil Ecol. 126, 113–120. doi: 10.1016/j.apsoil.2018.02.010

[B49] Diwakar M. C. MC D. MM P. (1980). Influence of some environmental and host factors on Pythium stalk rot of maize. Indian Phytopathology, 33.

[B50] Dodd I. C. Ruiz-Lozano J. M. (2012). Microbial enhancement of crop resource use efficiency. Curr. Opin. Biotechnol. 23, 236–242. doi: 10.1016/j.copbio.2011.09.005 21982722

[B51] Dovas C. I. Eythymiou K. Katis N. I. (2004). First report of Maize rough dwarf virus (MRDV) on maize crops in Greece. Plant Pathol. 53, 238. doi: 10.1111/j.0032-0862.2004.00973.x

[B52] Duvick D. N. (2005). The contribution of breeding to yield advances in maize (*Zea mays* L.). Adv. Agron. 86, 83–145. doi: 10.1016/S0065-2113(05)86002-X

[B53] Emamverdian A. Ding Y. Mokhberdoran F. Xie Y. (2015). Heavy metal stress and some mechanisms of plant defense response. Sci. World J., 756120. doi: 10.1155/2015/756120 PMC4321847 25688377

[B54] Ernst D. Kolenčík M. Šebesta M. Žitniak Čurná V. Qian Y. Straka V.. (2024). Enhancing maize yield and quality with metal-based nanoparticles without translocation risks: A brief field study. Plants 13, 1936. doi: 10.3390/plants13141936 39065463 PMC11280334

[B55] Exconde O. R. (1970). Philippine corn downy mildew. Indian Phytopathol. 23, 275–284.

[B56] Fang R. Lin J. Yao S. Wang Y. Wang J. Zhou C.. (2013). Promotion of plant growth, biological control and induced systemic resistance in maize by *Pseudomonas aurantiaca* JD37. Ann. Microbiol. 63, 1177–1185. doi: 10.1007/s13213-012-0576-7/

[B57] Farooq M. Hussain M. Wakeel A. Siddique K. H. (2015). Salt stress in maize: effects, resistance mechanisms, and management. A review. Agron. Sustain. Dev. 35, 461–481. doi: 10.1007/s13593-015-0287-0

[B58] Farooq M. Wahid A. Kobayashi N. S. M. A. Fujita D. B. S. M. A. Basra S. M. A. (2009). “Plant drought stress: effects, mechanisms and management,” in Sustainable agriculture (Springer, Dordrecht), 153–188.

[B59] Fornah A. Aula L. Omara P. Oyebiyi F. Dhillon J. Raun W. R. (2020). Effect of spacing, planting methods and nitrogen on maize grain yield. Commun. Soil Sci. Plant Anal. 51 (12), 1582–1589. doi: 10.1080/00103624.2020.1789163

[B60] Foyer C. H. Neukermans J. Queval G. Noctor G. Harbinson J. (2012). Photosynthetic control of electron transport and the regulation of gene expression. J. Exp. Bot. 63, 1637–1661. doi: 10.1093/jxb/ers013 22371324

[B61] Franco A. Buoso S. Zanin L. Pinton R. Tomasi N. (2023). Copper toxicity in maize: the severity of the stress is reduced depending on the applied fe-chelating agent. J. Plant Growth Regul. 42, 1567–1581. doi: 10.1007/s00344-022-10641-1

[B62] Frederiksen R. A. Renfro B. L. (1977). Global status of maize downy mildew. A. Rev. Phytopathol. 15, 249–275. doi: 10.1146/annurev.py.15.090177.001341

[B63] Frey T. J. Weldekidan T. Colbert T. Wolters P. J. C. C. Hawk J. A. (2011). Fitness evaluation of Rcg1, a locus that confers resistance to *Colletotrichum graminicola* (Ces.) GW Wils. using near-isogenic maize hybrids. Crop Sci. 51, 1551–1563. doi: 10.2135/cropsci2010.10.0613

[B64] Gedil M. Menkir A. (2019). An integrated molecular and conventional breeding scheme for enhancing genetic gain in maize in Africa. Front. Plant Sci. 10, 1430. doi: 10.3389/fpls.2019.01430 31781144 PMC6851238

[B65] Glick B. R. (2010). Using soil bacteria to facilitate phytoremediation. Biotechnol. Adv. 28, 367–374. doi: 10.1016/j.biotechadv.2010.02.001 20149857

[B66] Gong F. Wu X. Zhang H. Chen Y. Wang W. (2015). Making better maize plants for sustainable grain production in a changing climate. Front. Plant Sci. 6, 835. doi: 10.3389/fpls.2015.00835 26500671 PMC4593952

[B67] Gould F. Brown Z. S. Kuzma J. (2018). Wicked evolution: Can we address the sociobiological dilemma of pesticide resistance? Science 360 (6390), 728–732. doi: 10.1126/science.aar3780 29773742

[B68] Gorjanc G. Jenko J. Hearne S. J. Hickey J. M. (2016). Initiating maize pre-breeding programs using genomic selection to harness polygenic variation from landrace populations. BMC Genomics 17, 30. doi: 10.1186/s12864-015-2345-z 26732811 PMC4702314

[B69] Gould J. Devey M. Hasegawa O. Ulian E. C. Peterson G. Smith R. H. (1991). Transformation of *Zea mays* L. using *Agrobacterium tumefaciens* and the shoot apex. Plant Physiol. 95, 426–434. doi: 10.1104/pp.95.2.426 16668001 PMC1077548

[B70] Gupta A. Kaushal R. (2015). “Improving spam detection in online social networks,” in 2015 international conference on cognitive computing and information processing (CCIP) (IEEE), 1–6.

[B71] Gurung G. Manandhar K. L. Batsa B. K. (1985). “Efficacy of fungicides against head smut of maize,” in Proceedings of 12th Summer crops workshop, Rampur, Nepal, National Maize Development Program., 95–98.

[B72] Halford N. G. Curtis T. Y. Chen Z. Huang J. (2015). Effects of abiotic stress and crop management on cereal grain composition: implications for food quality and safety. J. Exp. Bot. 66, 1145–1156. doi: 10.1093/jxb/eru473 25428997 PMC4438447

[B73] Hansen G. Wright M. S. (1999). Recent advances in the transformation of plants. Trends Plant Sci. 4, 226–231. doi: 10.1016/S1360-1385(99)01412-0 10366879

[B74] Hashem A. Abd_Allah E. F. Alqarawi A. A. Al Huqail A. A. Egamberdieva D. Wirth S. (2016). Alleviation of cadmium stress in *Solanum lycopersicum* L. by arbuscular mycorrhizal fungi via induction of acquired systemic tolerance. Saudi J. Biol. Sci. 23, 272–281. doi: 10.1016/j.sjbs.2015.11.002 26981010 PMC4778590

[B75] Holišová V. Urban M. Kolenčík M. Němcová Y. Schröfel A. Peikertová P.. (2019). Biosilica-nanogold composite: Easy-to-prepare catalyst for soman degradation. Arabian J. Chem. 12, 262–271. doi: 10.1016/j.arabjc.2017.08.003

[B76] Hu S. Hu X. Hu J. Shang L. Dong G. Zeng D.. (2018). Xiaowei, a new rice germplasm for large-scale indoor research. Mol. Plant 11, 1418–1420. doi: 10.1016/j.molp.2018.08.003 30121299

[B77] Huang Y. Wang H. Zhu Y. Huang X. Li S. Wu X.. (2022). THP9 enhances seed protein content and nitrogen-use efficiency in maize. Nature 612 (7939), 292–300. doi: 10.1111/nph.18733 36385527

[B78] Hudson A. I. Odell S. G. Dubreuil P. Tixier M. H. Praud S. Runcie D. E.. (2022). Analysis of genotype-by-environment interactions in a maize mapping population. G3 (Bethesda) 12, jkac013. doi: 10.1093/g3journal/jkac013 35134181 PMC8895993

[B79] Hussain H. A. Men S. Hussain S. Chen Y. Ali S. Zhang S.. (2019). Interactive effects of drought and heat stresses on morpho-physiological attributes, yield, nutrient uptake and oxidative status in maize hybrids. Sci. Rep. 9, 1–12. doi: 10.1038/s41598-019-40362-7 30846745 PMC6405865

[B80] Icoz I. Stotzky G. (2008). Cry3Bb1 protein from Bacillus thuringiensis in root exudates and biomass of transgenic corn does not persist in soil. Transgenic Res. 17, 609–620. doi: 10.1007/s11248-007-9133-8 17851773

[B81] Iqbal S. Hussain S. Qayyaum M. A. Ashraf M. (2020). The response of maize physiology under salinity stress and its coping strategies. Plant Stress Physiol., 1–25. doi: 10.5772/intechopen.92213

[B82] Iqbal S. Iqbal M. A. Chunjia Li C. Iqbal A. Abbas R. N. (2023). Overviewing drought and heat stress amelioration-from plant responses to microbe-mediated mitigation. Sustainability 15, 1671. doi: 10.3390/su15021671

[B83] Ishida Y. Saito H. Ohta S. Hiei Y. Komari T. Kumashiro T. (1996). High efficiency transformation of maize (*Zea mays* L.) mediated by Agrobacterium tumefaciens. Nat. Biotechnol. 14, 745–750. doi: 10.1038/nbt0696-745 9630983

[B84] Islam M. S. Islam M. R. Hasan M. K. Hafeez A. G. Chowdhury M. K. Pramanik M. H.. (2024). Salinity stress in maize: consequences, tolerance mechanisms, and management strategies. OBM Genet. 8, 232. doi: 10.21926/obm.genet.2402232

[B85] James C. (2003). Global review of commercialized transgenic crops: 2002 feature: Bt maize Vol. 29 (Ithaca, NY: ISAAA).

[B86] Janda T. Szalai G. Tari I. Paldi E. (1999). Hydroponic treatment with salicylic acid decreases the effects of chilling injury in maize (*Zea mays* L.) plants. Planta 208, 175–180. doi: 10.1007/s004250050547

[B87] Jeffers D. (2004). Maize diseases: a *guide for field identification* (Mexico: Cimmyt).

[B88] Ji W. He H. Zhao S. Yan S. Fu D. (2010). Identification of physiological races of *Setosphaeria turcica* in maize-producing region of Heilongjiang. J. Maize Sci. 18, 128–134.

[B89] Jiang S. Zhang D. Wang L. Pan J. Liu Y. Kong X.. (2013). A maize calcium-dependent protein kinase gene, ZmCPK4, positively regulated abscisic acid signaling and enhanced drought stress tolerance in transgenic Arabidopsis. Plant Physiol. Biochem. 71, 112–120. doi: 10.1016/j.plaphy.2013.07.004 23911729

[B90] Jing Y. X. Yan J. L. He H. D. Yang D. J. Xiao L. Zhong T.. (2014). Characterization of bacteria in the rhizosphere soils of *Polygonum pubescens* and their potential in promoting growth and Cd, Pb, Zn uptake by *Brassica napus* . Int. J. phytoremed. 16, 321–333. doi: 10.1080/15226514.2013.773283 24912234

[B91] Kasim W. A. Gaafar R. M. Abou-Ali R. M. Omar M. N. Hewait H. M. (2016). Effect of biofilm forming plant growth promoting rhizobacteria on salinity tolerance in barley. Ann. Agric. Sci. 61, 217–227. doi: 10.1016/j.aoas.2016.07.003

[B92] Kaur G. Vikal Y. Kaur L. Kalia A. Mittal A. Kaur D.. (2021). Elucidating the morpho-physiological adaptations and molecular responses under long-term waterlogging stress in maize through gene expression analysis. Plant Sci. 304, 110823. doi: 10.1016/j.plantsci.2021.110823 33568312

[B93] Kenganal M. Patil M. B. Nimbaragi Y. (2017). Management of stalk rot of maize caused by *Fusarium moniliforme* (Sheldon). Int. J. Curr. Microbiol. Appl. Sci. 6, 3546–3552. doi: 10.20546/ijcmas.2017.609.436

[B94] Khadka B. B. Shah S. M. (1967). Preliminary list of plant diseases recorded in Nepal. Nepalese J. Agric. 2, 47–76.

[B95] Khosravi A. R. Mansouri M. Bahonar A. R. Shokri H. (2007). Mycoflora of maize harvested from Iran and imported maize. Pakistan J. Biol. Sciences: PJBS 10, 4432–4437. doi: 10.3923/pjbs.2007.4432.4437 19093507

[B96] Kim K. H. Lee B. M. (2023). Effects of climate change and drought tolerance on maize growth. Plants 12, 3548. doi: 10.3390/plants12203548 37896012 PMC10610049

[B97] Kimotho R. N. Baillo E. H. Zhang Z. (2019). Transcription factors involved in abiotic stress responses in Maize (*Zea mays* L.) and their roles in enhanced productivity in the post genomics era. PeerJ 7, e7211. doi: 10.7717/peerj.7211 31328030 PMC6622165

[B98] Klich M. A. (2007). *Aspergillus flavus*: the major producer of aflatoxin. Mol. Plant Pathol. 8, 713–722. doi: 10.1111/j.1364-3703.2007.00436.x 20507532

[B99] Kloepper J. W. (1978). “Plant growth-promoting rhizobacteria on radishes,” in Proc. of the 4th Internet. Conf. on Plant Pathogenic Bacter, Angers, France, 1978, Vol. 2, 879–882 (INRA, Angers, France: Station de Pathologie Vegetale et Phytobacteriologie, INRA).

[B100] Koini M. A. Alvey L. Allen T. Tilley C. A. Harberd N. P. Whitelam G. C.. (2009). High temperature-mediated adaptations in plant architecture require the bHLH transcription factor PIF4. Curr. Biol. 19, 408–413. doi: 10.1016/j.cub.2009.01.046 19249207

[B101] Kolenčík M. Nemček L. Šebesta M. Urík M. Ernst D. Kratošová G.. (2021). Effect of TiO 2 as plant growth-stimulating nanomaterial on crop production. Plant Responses to Nanomater.: Recent Intervent. Physiol. Biochem. Responses, 129–144. doi: 10.1007/978-3-030-36740-4_5

[B102] Krishnamoorthy R. Kim K. Subramanian P. Senthilkumar M. Anandham R. Sa T. (2016). Arbuscular mycorrhizal fungi and associated bacteria isolated from salt-affected soil enhances the tolerance of maize to salinity in coastal reclamation soil. Agricult. Ecosyst. Environ. 231, 233–239. doi: 10.1016/j.agee.2016.05.037

[B103] Kuan K. B. Othman R. Rahim A. K. Shamsuddin Z. H. (2016). Plant growth-promoting rhizobacteria inoculation to enhance vegetative growth, nitrogen fixation and nitrogen remobilisation of maize under greenhouse conditions. PloS One 11, e0152478. doi: 10.1371/journal.pone.0152478 27011317 PMC4807084

[B104] Kumar A. (2015). Genetic diversity of Erwinia chrysanthemi pv. zeae causing bacterial stalk rot of maize and its management. Ludhiana: Department of Plant Pathology, PAU.

[B105] Kumari M. Dass S. Vimala Y. Arora P. (2004). Physiological parameters governing drought tolerance in maize. Indian J. Plant Physiol. 9, 203–207.

[B106] Kumari M. Naidu S. Kumari B. Singh I. K. Singh A. (2023). Comparative transcriptome analysis of Zea mays upon mechanical wounding. Mol. Biol. Rep. 50, 5319–5343. doi: 10.1007/s11033-023-08429-x 37155015

[B107] Kushalappa A. C. Hegde R. K. (1970). Studies on maize rust (P. sorghi) in Mysore state III: Prevalence and severity on maize varieties and impact on yield. Plant Dis. Rep. 54, 15–20.

[B108] Lai Y. R. Lin P. Y. Chen C. Y. Huang C. J. (2016). Feasible management of southern corn leaf blight via induction of systemic resistance by Bacillus cereus C1L in combination with reduced use of dithiocarbamate fungicides. Plant Pathol. J. 32, 481. doi: 10.5423/PPJ.OA.02.2016.0044 27721698 PMC5051567

[B109] Lal S. Saxena S. C. Upadhyay N. (1980). Control of brown stripe downy mildew of maize by Metalaxyl. Seed 2, 5–018. doi: 10.1094/PD-64-874

[B110] Li H. Wang H. L. Du J. Du G. Zhan J. C. Huang W. D. (2010). Trehalose protects wine yeast against oxidation under thermal stress. World J. Microbiol. Biotechnol. 26, 969–976. doi: 10.1007/s11274-009-0258-1

[B111] Li K. Xu C. Zhang J. (2011). Proteome profile of maize (*Zea mays* L.) leaf tissue at the flowering stage after long-term adjustment to rice black-streaked dwarf virus infection. Gene 485, 106–113. doi: 10.1016/j.gene.2011.06.016 21708230

[B112] Li W. C. Ye Z. H. Wong M. H. (2007). Effects of bacteria on enhanced metal uptake of the Cd/Zn-hyperaccumulating plant, Sedum alfredii. J. Exp. Bot. 58, 4173–4182. doi: 10.1093/jxb/erm274 18039737

[B113] Liang B.-W. Li C. Bai T.-H. Wang P. (2023). Editorial: Nutrient use efficiency of plants under abiotic stress. Front. Plant Sci. 14. doi: 10.3389/fpls.2023.1179842 PMC10250746 37304712

[B114] Liang C. Tian J. Liao H. (2013). Proteomics dissection of plant responses to mineral nutrient deficiency. Proteomics 13, 624–636. doi: 10.1002/pmic.201200263 23193087

[B115] Lim J. A. Yaacob J. S. Mohd Rasli S. R. A. Eyahmalay J. E. El Enshasy H. A. Zakaria M. R. S. (2023). Mitigating the repercussions of climate change on diseases affecting important crop commodities in Southeast Asia, for food security and environmental sustainability—A review. Front. Sustain. Food Syst. 6, 1030540. doi: 10.3389/fsufs.2022.1030540

[B116] Liu R. Lal R. (2015). Potentials of engineered nanoparticles as fertilizers for increasing agronomic productions. Sci. total Environ. 514, 131–139. doi: 10.1016/j.scitotenv.2015.01.104 25659311

[B117] Liu X. Chai J. Zhang Y. (2022). Halotolerant rhizobacteria mitigate the effects of salinity stress on maize growth by secreting exopolysaccharides. Environ. Exp. Bot. 204, 204–105098. doi: 10.1016/j.envexpbot.2022.105098

[B118] Lobell D. B. Roberts M. J. Schlenker W. Braun N. Little B. B. Rejesus R. M.. (2014). Greater sensitivity to drought accompanies maize yield increase in the US Midwest. Science 344, 516–519. doi: 10.1126/science.1251423 24786079

[B119] Lobo L. L. B. dos Santos R. M. Rigobelo E. C. (2019). Promotion of maize growth using endophytic bacteria under greenhouse and field conditions. Aust. J. Crop Sci. 13, 2067–2074. doi: 10.21475/ajcs

[B120] Lodha T. D. Hembram P. Nitile Tep J. B. (2013). Proteomics: a successful approach to understand the molecular mechanism of plant-pathogen interaction. Am. J. Plant Sci. 4 (6), 15. doi: 10.4236/ajps.2013.46149

[B121] Magar M. M. Parajuli A. Sah B. P. Shrestha J. (2019). Effect of PEG induced drought stress on germination and seedling traits of maize (*Zea mays* L.) lines. Türk Tarım ve Doğa Bilimleri Dergisi 6, 196–205. doi: 10.30910/turkjans.556607

[B122] Manandhar K. L. (1972). Study on downy mildew disease of maize under Rampur condition. Proc. Maize seminar held June, 20–22.

[B123] Manandhar K. L. (1975). Second supplementary list of plant disease in Nepal (FAO Bulletin technical Document No. 97).

[B124] Mandal H. R. Katel S. Subedi S. Shrestha J. (2021). Plant Parasitic Nematodes and their management in crop production: a review. J. Agric. Natural Resour. 4, 327–338. doi: 10.3126/janr.v4i2.33950

[B125] Mao H. Wang H. Liu S. Li Z. Yang X. Yan J.. (2015). A transposable element in a NAC gene is associated with drought tolerance in maize seedlings. Nat. Commun. 6, 1–13. doi: 10.1038/ncomms9326 PMC4595727 26387805

[B126] Marks B. B. Megías M. Ollero F. J. Nogueira M. A. Araujo R. S. Hungria M. (2015). Maize growth promotion by inoculation with *Azospirillum brasilense* and metabolites of Rhizobium tropici enriched on lipo-chitooligosaccharides (LCOs). Amb Express 5, 1–11. doi: 10.1186/s13568-015-0154-z 26567001 PMC4644132

[B127] Martinez-Cisneros B. A. Juarez-Lopez G. Valencia-Torres N. Duran-Peralta E. Mezzalama M. (2014). First report of bacterial stalk rot of maize caused by Dickeya zeae in Mexico. Plant Dis. 98, 1267–1267. doi: 10.1094/PDIS-02-14-0198-PDN 30699660

[B128] Martre P. Quilot-Turion B. Luquet D. Memmah M. M. O. S. Chenu K. Debaeke P. (2015). Model-assisted phenotyping and ideotype design. Crop Physiol., 349–373. doi: 10.1016/B978-0-12-417104-6.00014-5

[B129] Mendel J. M. (2017). Uncertain rule-based fuzzy systems. Introduction New Dir., 684. doi: 10.1007/978-3-319-51370-6

[B130] Mengesha W. A. Menkir A. Unakchukwu N. Meseka S. Farinola A. Girma G.. (2017). Genetic diversity of tropical maize inbred lines combining resistance to *Striga hermonthica* with drought tolerance using SNP markers. Plant Breed. 136, 338–343. doi: 10.1111/pbr.12479

[B131] Menkir A. Ingelbrecht I. (2007). Testcross performance and diversity analysis of white maize lines derived from backcrosses containing exotic germplasm. Euphytica 155, 417–428. doi: 10.1007/s10681-006-9344-8

[B132] Menkir A. Kling J. G. Anjorin B. Ladejobi F. Gedil M. (2015). Evaluating testcross performance and genetic divergence of lines derived from reciprocal tropical maize composites. Maydica 60, 1–10. Available online at: http://www.maydica.org/articles/60_2_14.pdf.

[B133] Menkir A. Olowolafe M. O. Ingelbrecht I. Fawole I. Badu-Apraku B. Vroh B. I. (2006). Assessment of testcross performance and genetic diversity of yellow endosperm maize lines derived from adapted× exotic backcrosses. Theor. Appl. Genet. 113, 90–99. doi: 10.1007/s00122-006-0275-5 16614832

[B134] Meseka S. Menkir A. Bossey B. Mengesha W. (2018). Performance assessment of drought tolerant maize hybrids under combined drought and heat stress. Agronomy 8, 274. doi: 10.3390/agronomy8120274 33304638 PMC7672364

[B135] Mesterhazy A. Toth T. E. Szel S. Varga M. Toth B. (2020). Resistance of maize hybrids to *Fusarium graminearum, F. culmorum*, and *F. verticillioides* ear rots with toothpick and silk channel inoculation, as well as their toxin production. Agronomy 10, 1283. doi: 10.3390/agronomy10091283

[B136] Metcalf R. L. (1986). Coevolutionary adaptations of rootworm beetles (Coleoptera: Chrysomelidae) to cucurbitacins. J. Chem. Ecol. 12, 1109–1124. doi: 10.1007/BF01638999 24307050

[B137] Miao Z. Han Z. Zhang T. Chen S. Ma C. (2017). A systems approach to a spatio-temporal understanding of the drought stress response in maize. Sci. Rep. 7, 1–14. doi: 10.1038/s41598-017-06929-y 28747711 PMC5529502

[B138] Mohammadi M. Anoop V. Gleddie S. Harris L. J. (2011). Proteomic profiling of two maize inbreds during early gibberella ear rot infection. Proteomics 11, 3675–3684. doi: 10.1002/pmic.201100177 21751381

[B139] Montañez A. Blanco A. R. Barlocco C. Beracochea M. Sicardi M. (2012). Characterization of cultivable putative endophytic plant growth promoting bacteria associated with maize cultivars (*Zea mays* L.) and their inoculation effects in *vitro* . Appl. Soil Ecol. 58, 21–28. doi: 10.1016/j.apsoil.2012.02.009

[B140] Muhammad I. Yang L. Ahmad S. Farooq S. (2023). Melatonin-priming enhances maize seedling drought tolerance by regulating the antioxidant defense system. Plant Physiol. 191, 2301–2315. doi: 10.1093/plphys/kiad027 36660817 PMC10069899

[B141] Munkvold G. P. Desjardins A. E. (1997). Fumonisins in maize: can we reduce their occurrence? Plant Dis. 81, 556–565. doi: 10.1094/PDIS.1997.81.6.556 30861834

[B142] Nadeem S. M. Zahir Z. A. Naveed M. Arshad M. (2009). Rhizobacteria containing ACC-deaminase confer salt tolerance in maize grown on salt-affected fields. Can. J. Microbiol. 55, 1302–1309. doi: 10.1139/W09-092 19940939

[B143] Naik K. Mishra S. Srichandan H. Singh P. K. Sarangi P. K. (2019). Plant growth promoting microbes: Potential link to sustainable agriculture and environment. Biocatal. Agric. Biotechnol. 21, 101326. doi: 10.1016/j.bcab.2019.101326

[B144] Naseem H. Bano A. (2014). Role of plant growth-promoting rhizobacteria and their exopolysaccharide in drought tolerance of maize. J. Plant Interact. 9, 689–701. doi: 10.1080/17429145.2014.902125

[B145] Ngara R. Ndimba R. Borch-Jensen J. Jensen O. N. Ndimba B. (2012). Identification and profiling of salinity stress-responsive proteins in sorghum bicolor seedlings. J. Proteomics 75 (13), 4139–4150. doi: 10.1016/j.jprot.2012.05.038 22652490

[B146] Nur H. B. Michaela L. Made S. Josh V. V. Peter A. Amy I.. (2020). Meeting the food security challenge for nine billion people in 2050: What impact on forests? Global Environ. Change 65, 102195. doi: 10.1016/j.gloenvcha.2020.102056

[B147] Odell S. G. Hudson A. I. Praud S. Dubreuil P. Tixier M. H. Ross-Ibarra J.. (2022). Modeling allelic diversity of multi-parent mapping populations affects detection of quantitative trait loci. G3 (Bethesda). 12 (3), 1–14. doi: 10.1093/g3journal/jkac011 PMC8895984 35100382

[B148] Ortiz N. Armada E. Duque E. Roldán A. Azcón R. (2015). Contribution of arbuscular mycorrhizal fungi and/or bacteria to enhancing plant drought tolerance under natural soil conditions: effectiveness of autochthonous or allochthonous strains. J. Plant Physiol. 174, 87–96. doi: 10.1016/j.jplph.2014.08.019 25462971

[B149] Osmolovskaya N. Shumilina J. Kim A. Didio A. Grishina T. Bilova T.. (2018). Methodology of drought stress research: Experimental setup and physiological characterization. Int. J. Mol. Sci. 19, 4089. doi: 10.3390/ijms19124089 30563000 PMC6321153

[B150] Pal K. K. Tilak K. R. Saxcna A. K. Dey R. Singh C. S. (2001). Suppression of maize root diseases caused by *Macrophomina phaseolina, Fusarium moniliforme* and *Fusarium graminearum* by plant growth promoting rhizobacteria. Microbiol. Res. 156, 209–223. doi: 10.1078/0944-5013-00103 11716210

[B151] Pareek A. Sopory S. K. Bohnert H. J. Govindjee (2010). Abiotic stress adaptation in plants: physiological, molecular and genomic foundation. Berlin: Springer 10, 978–990.

[B152] Payak M. M. Sharma R. C. Menon K. (1979). Sorghum downy mildew of maizephysiologic specialization and resistant sources. Rev. la Facllitad Agronom., 177–187.

[B153] Pechanova O. Pechan T. Williams W. P. Luthe D. S. (2011). Proteomic analysis of the maize rachis: potential roles of constitutive and induced proteins in resistance to *Aspergillus flavus* infection and aflatoxin accumulation. Proteomics 11, 114–127. doi: 10.1002/pmic.201000368 21182199

[B154] Perera M. F. Filippone M. P. Ramallo C. J. Cuenya M. I. Gracia M. L. Ploper L. D.. (2009). Genetic diversity among viruses associated with sugarcane mosaic disease in Tucumán, Argentina. Phytopathology 99, 38–49. doi: 10.1094/PHYTO-99-1-0038 19055433

[B155] Pérez-Montaño F. Jiménez-Guerrero I. Sánchez-Matamoros R. C. López-Baena F. J. Ollero F. J. Rodríguez-Carvajal M. A.. (2013). Rice and bean AHL-mimic quorum-sensing signals specifically interfere with the capacity to form biofilms by plant-associated bacteria. Res. Microbiol. 164, 749–760. doi: 10.1016/j.resmic.2013.04.001 23583723

[B156] Popp J. Peto K. Nagy J. (2013). Pesticide productivity and food security. A review. Agron. Sustain. Dev. 33, 243–255. doi: 10.1007/s13593-012-0105-x

[B157] Prapagdee B. Chanprasert M. Mongkolsuk S. (2013). Bioaugmentation with cadmium-resistant plant growth-promoting rhizobacteria to assist cadmium phytoextraction by *Helianthus annuus* . Chemosphere 92, 659–666. doi: 10.1016/j.chemosphere.2013.01.082 23478127

[B158] Prasad H. H. (1930). A bacterial stalk rot of maize. Indian J. Agr. Sci. 25, 72. Available online at: https://www.cabidigitallibrary.org/doi/full/10.5555/19301100742.

[B159] Puente M. E. Li C. Y. Bashan Y. (2004). Microbial populations and activities in the rhizoplane of rock-weathering desert plants. II. Growth promotion of cactus seedlings. Plant Biol. 6, 643–650. doi: 10.1055/s-2004-821101 15375736

[B160] Qian J. Li D. Zhan G. Zhang L. Su W. Gao P. (2012). Simultaneous biodegradation of Ni–citrate complexes and removal of nickel from solutions by Pseudomonas alcaliphila. Biores. Technol. 116, 66–73. doi: 10.1016/j.biortech.2012.04.017 22609657

[B161] Qiao M. Hong C. Jiao Y. Hou S. Gao H. (2024). Impacts of drought on photosynthesis in major food crops and the related mechanisms of plant responses to drought. Plants 13, 1808. doi: 10.3390/plants13131808 38999648 PMC11243883

[B162] Rahman S. Ahmad I. Nafees M. (2023). Mitigation of heavy metal stress in maize (*Zea mays* L.) through application of silicon nanoparticles. Biocatal. Agric. Biotechnol. 50, 50–102757. doi: 10.1016/j.bcab.2023.102757

[B163] Raza A. Razzaq A. Mehmood S. S. Zou X. Zhang X. Lv Y.. (2019). Impact of climate change on crops adaptation and strategies to tackle its outcome: A review. Plants 8, 34. doi: 10.3390/plants8020034 30704089 PMC6409995

[B164] Riva-Roveda L. Escale B. Giauffret C. Périlleux C. (2016). Maize plants can enter a standby mode to cope with chilling stress. BMC Plant Biol. 16, 212. doi: 10.1186/s12870-016-0909-y 27716066 PMC5050578

[B165] Roberts M. J. (2006). The value of plant disease early-warning systems: a *case study of USDA’s soybean rust coordinated framework* (No. 18) (USDA: Economic Research Service).

[B166] Rorison I. H. (1986). The response of plants to acid soils. Experientia 42, 357–362. doi: 10.1007/BF02118616

[B167] Sade N. Rubio-Wilhelmi M. Umnajkitikorn K. Blumwald E. (2018). Stress-induced senescence and plant tolerance to abiotic stress. J. Exp. Bot. 69, 845–853. doi: 10.1093/jxb/erx235 28992323

[B168] Sadoma M. T. El-Sayed A. B. B. El-Moghazy S. M. (2011). Biological control of downy mildew disease of maize caused by *Peronosclerospora sorghi* using certain biocontrol agents alone or in combination. J. Agric. Res. 37, 1–11.

[B169] Samson R. Legendre J. B. Christen R. Fischer-Le Saux M. Achouak W. Gardan L. (2005). Transfer of *Pectobacterium chrysanthemi* and *Brenneria paradisiaca* to the genus Dickeya gen. Int. J. System. Evolution. Microbiol. 55, 1415–1427. doi: 10.1099/ijs.0.02791-0 16014461

[B170] Sandhya V. S. Ali S. K. Z. Grover M. Reddy G. Venkateswarlu B. S. (2009). Alleviation of drought stress effects in sunflower seedlings by the exopolysaccharides producing *Pseudomonas putida* strain GAP-P45. Biol. fertil. soils 46, 17–26. doi: 10.1007/s00374-009-0401-z

[B171] Scafaro A. P. Posch B. C. Evans J. R. Farquhar G. D. Atkin O. K. (2023). Rubisco deactivation and chloroplast electron transport rates co-limit photosynthesis above optimal leaf temperature in terrestrial plants. Nat. Commun. 14, 2820. doi: 10.1038/s41467-023-38496-4 37198175 PMC10192301

[B172] Schilmiller A. L. Howe G. A. (2005). Systemic signaling in the wound response. Curr. Opin. Plant Biol. 8, 369–377. doi: 10.1016/j.pbi.2005.05.008 15939667

[B173] Šebesta M. Ďurišová Ľ. Ernst D. Kšiňan S. Illa R. Sunil B. R.. (2022). “Foliar application of metallic nanoparticles on crops under field conditions,” in Plant and Nanoparticles (Springer Nature Singapore, Singapore), 171–215.

[B174] Shah S. M. (1976). Downy mildew of maize in Nepal. Agric. Natural Resour. 10, 137–142.

[B175] Shah M. Tuladhar K. (1971). “Downy mildew diseases of maize in Nepal,” in Proceedings 7th International Asian corn improvement workshop, Los Bahos, Philippines. 88–94.

[B176] Shahzad A. Anam A. S. (2023). Heavy metals mitigation and growth promoting effect of endophytic *Agrococcus terreus* (MW 979614) in maize plants under zinc and nickel contaminated soil. Front. Microbiol. 14. doi: 10.3389/fmicb.2023.1255921 PMC10668838 38029198

[B177] Sharma H. C. Vidyasagar P. Abraham C. V. Nwanze K. F. (1993). Effect of cytoplasmic male-sterility in sorghum on host plant interaction with sorghum midge, contarinia sorghicola. Euphytica 74, 35–39. doi: 10.1007/BF00033764

[B178] Sharma R. C. Payak M. M. Shankerlingam S. Laxminarayan C. (1982). A comparison of two methods of estimating yield losses in maize caused by common rust. Indian Phytopathol. 35.1, 18–20.

[B179] Sharma P. Nita Lakra N. Lakra N. Goyal A. (2023). Drought and heat stress mediated activation of lipid signaling in plants: a critical review. Front. Plant Sci. Sec. Plant Abiot. Stress. 14. doi: 10.3389/fpls.2023.1216835 PMC10450635 37636093

[B180] Sharma R. C. Payak M. M. (1979). Resistance to common rust of maize in India. Maize Genet. Coop. Newslett. 53, 665–666.

[B181] Shen Y. Zhang Y. Chen J. Lin H. Zhao M. Peng H.. (2013). Genome expression profile analysis reveals important transcripts in maize roots responding to the stress of heavy metal Pb. Physiol. Plant. 147, 270–282. doi: 10.1111/j.1399-3054.2012.01670.x 22747913

[B182] Silva E. D. Ribeiro R. V. Ferreira-Silva S. L. Viégas R. A. Silveira J. A. G. (2010). Comparative effects of salinity and water stress on photosynthesis, water relations and growth of *Jatropha curcas* plants. J. Arid Environ. 74, 1130–1137. doi: 10.1016/j.jaridenv.2010.05.036

[B183] Singh M. V. Prakash V. Singh B. H. A. G. W. A. N. Shahi H. N. (2014). Response of maize hybrids to integrated nutrient management. Haryana J. Agron. 30 (1), 65–69.

[B184] Singh R. P. Chitara M. K. Chauhan S. (2020). Bacterial stalk rot of maize and their management. Indian Farmers Digest 53, 10–11.

[B185] Singh A. Pandey A. Pandey S. Lal D. Chauhan D. Aparma. (2023). Drought stress in maize: stress perception to molecular response and strategies for its improvement. Funct. Integr. Genomics 23, 296. doi: 10.1007/s10142-023-01226-6 37697159

[B186] Sitara U. Akhter S. (2007). Efficacy of fungicides, sodium hypochloritc and neem seed powder to control seed borne pathogens of maize. Pakistan J. Bot. 39, 285. doi: 10.5555/20073105116

[B187] Subedi S. (2015). A review on important maize diseases and their management in Nepal. J. Maize Res. Develop. 1 (1), 28–52. doi: 10.3126/jmrd.v1i1.14242

[B188] Subhashini D. V. (2015). Growth promotion and increased potassium uptake of tobacco by potassium-mobilizing bacterium *Frateuria aurantia* grown at different potassium levels in vertisols. Commun. Soil Sci. Plant Anal. 46, 210–220. doi: 10.1080/00103624.2014.967860

[B189] Tabassum B. Khan A. Tariq M. Ramzan M. Khan M. S. I. Shahid N.. (2017). Bottlenecks in commercialisation and future prospects of PGPR. Appl. Soil Ecol. 121, 102–117. doi: 10.1016/j.apsoil.2017.09.030

[B190] Takeda S. Matsuoka M. (2008). Genetic approaches to crop improvement: responding to environmental and population changes. Nat. Rev. Genet. 9, 444–457. doi: 10.1038/nrg2342 18475268

[B191] Tang A. Haruna A. O. Muhamad N. Majid A. Jalloh M. B. (2020). Effects of selected functional bacteria on maize growth and nutrient use efficiency. Microorganisms 8, 854. doi: 10.3390/microorganisms8060854 32517011 PMC7356773

[B192] Thind B. S. Payak M. M. (1978). Evaluation of maize germplasm and estimation of losses to Erwinia stalk rot. Plant Dis. Rep. 62, 319–323. doi: 10.5555/19781345718

[B193] Thomashow M. F. (2010). Molecular basis of plant cold acclimation: insights gained from studying the CBF cold response pathway. Plant Physiol. 154, 571–577. doi: 10.1104/pp.110.161794 20921187 PMC2948992

[B194] Tian L. Li J. Bi W. Zuo S. Li L. Li W.. (2019). Effects of waterlogging stress at different growth stages on the photosynthetic characteristics and grain yield of spring maize (Zea mays L.) Under field conditions. Agric. Water Manage. 218, 250–258. doi: 10.1016/j.agwat.2019.03.054

[B195] Tigar B. J. Osborne P. E. Key G. E. Flores-S M. E. Vazquez-A M. (1994). Insect pests associated with rural maize stores in Mexico with particular reference to *Prostephanus truncatus* (*Coleoptera*: Bostrichidae). J. Stored Prod. Res. 30, 267–281. doi: 10.1016/S0022-474X(94)90319-0

[B196] Tiwari Y. K. Yadav S. K. (2019). High temperature stress tolerance in maize (Zea mays L.): physiological and molecular mechanisms. J. Plant Biol. 62, 93–102. doi: 10.1007/s12374-018-0350-x

[B197] Tubiello F. N. Mayo R. Salvatore M. (2009). Agriculture and greenhouse gases: FAO’s approach to addressing the unique challenges faced by agricultural statisticians. (Rome, Italy, Viale delle Terme di Caracalla).

[B198] Turrini A. Sbrana C. Giovannetti M. (2015). Belowground environmental effects of transgenic crops: a soil microbial perspective. Res. Microbiol. 166, 121–131. doi: 10.1016/j.resmic.2015.02.006 25728596

[B199] Turrini A. Sbrana C. Pitto L. Ruffini Castiglione M. Giorgetti L. Briganti R.. (2004). The antifungal Dm-AMP1 protein from *Dahlia merckii* expressed in *Solanum melongena* is released in root exudates and differentially affects pathogenic fungi and mycorrhizal symbiosis. New Phytol. 163, 393–403. doi: 10.1111/j.1469-8137.2004.01107.x 33873617

[B200] Ullstrup A. J. (1972). Impacts of the southern corn leaf blight epidemics of 1970–1971. Annu. Rev. Phytopathol. 10, 37–50. doi: 10.1146/annurev.py.10.090172.000345

[B201] Uppal B. N. Desai M. K. (1932). Two new hosts of the downy mildew of Sorghum in Bombay. Phytopathology 22, 587–594. doi: 10.5555/19321101362

[B202] Utpal D. Ritika B. (2015). Integrated disease management strategy of common rust of maize incited by *Puccinia sorghi* Schw. Afr. J. Microbiol. Res. 9, 1345–1351. doi: 10.5897/AJMR

[B203] van Egmond H. P. Schothorst R. C. Jonker M. A. (2007). Regulations relating to mycotoxins in food. Anal. bioanal. Chem. 389, 147–157. doi: 10.1007/s00216-007-1317-9 17508207

[B204] Vardharajula S. Zulfikar Ali S. Grover M. Reddy G. Bandi V. (2011). Drought-tolerant plant growth promoting Bacillus spp.: effect on growth, osmolytes, and antioxidant status of maize under drought stress. J. Plant Interact. 6, 1–14. doi: 10.1080/17429145.2010.535178

[B205] Vennam R. R. Bheemanahalli R. Reddy K. R. Dhillon J. Zhang X. Adeli A. (2024). Early-season maize responses to salt stress: Morpho-physiological, leaf reflectance, and mineral composition. J. Agric. Food Res. 15, 100994. doi: 10.1016/j.jafr.2024.100994

[B206] Verma S. Kuila A. (2019). Bioremediation of heavy metals by microbial process. Environ. Technol. Innovation 14, 100369. doi: 10.1016/j.eti.2019.100369

[B207] Vijayaraghavan K. Yun Y. S. (2008). Bacterial biosorbents and biosorption. Biotechnol. Adv. 26, 266–291. doi: 10.1016/j.biotechadv.2008.02.002 18353595

[B208] Wang N. Yuan Y. Wang H. Yu D. Liu Y. Zhang A.. (2020). Applications of genotyping-by-sequencing (GBS) in maize genetics and breeding. Sci. Rep. 10, 16308. doi: 10.1038/s41598-020-73321-8 33004874 PMC7530987

[B209] Wang P. Chen Z. (2013). Traditional Chinese medicine ZHENG and Omics convergence: a systems approach to post-genomics medicine in a global world. Omics: J. Integr. Biol. 17, 451–459. doi: 10.1089/omi.2012.0057 23837436

[B210] Waqas M. A. Wang X. Zafar S. A. Noor M. A. Hussain H. A. Azher Nawaz M.. (2021). Thermal stresses in maize: effects and management strategies. Plants 10, 293. doi: 10.3390/plants10020293 33557079 PMC7913793

[B211] Warburton M. L. Reif J. C. Frisch M. Bohn M. Bedoya C. Xia X. C.. (2008). Genetic diversity in CIMMYT nontemperate maize germplasm: landraces, open pollinated varieties, and inbred lines. Crop Sci. 48, 617–624. doi: 10.2135/cropsci2007.02.0103

[B212] Wei J. K. Liu K. M. Chen J. P. Luo P. C. Stadelmann O. L. (1988). Pathological and physiological identification of race C of *Bipolaris maydis* in China. Phytopathology 78, 550–554. doi: 10.1094/Phyto-78-550

[B213] Wu F. Miller J. D. Casman E. A. (2004). The economic impact of Bt corn resulting from mycotoxin reduction. J. Toxicol.: Toxin Rev. 23, 397–424. doi: 10.1081/TXR-200027872

[B214] Wu F. Shu J. Jin W. (2014). Identification and validation of miRNAs associated with the resistance of maize (*Zea mays* L.) to *Exserohilum turcicum* . PloS One 9, e87251. doi: 10.1371/journal.pone.0087251 24489881 PMC3906166

[B215] Wu L. Han Z. Wang S. Wang X. Sun A. Zu X.. (2013). Comparative proteomic analysis of the plant–virus interaction in resistant and susceptible ecotypes of maize infected with sugarcane mosaic virus. J. Proteomics 89, 124–140. doi: 10.1016/j.jprot.2013.06.005 23770298

[B216] Xie T. Gu W. Meng Y. Li J. Li L. Wang Y.. (2017). Exogenous DCPTA ameliorates simulated drought conditions by improving the growth and photosynthetic capacity of maize seedlings. Sci. Rep. 7, 1–13. doi: 10.1038/s41598-017-12977-1 28978944 PMC5627246

[B217] Xu D. L. Park J. W. Mirkov T. E. Zhou G. H. (2008). Viruses causing mosaic disease in sugarcane and their genetic diversity in southern China. Arch. Virol. 153, 1031–1039. doi: 10.1007/s00705-008-0072-3 18438601

[B218] Xu G. Fan X. Miller A. J. (2012). Plant nitrogen assimilation and use efficiency. Annu. Rev. Plant Biol. 63, 153–182. doi: 10.1146/annurev-arplant-042811-105532 22224450

[B219] Xu M. L. Melchinger A. E. Lübberstedt T. (1999). Species-specific detection of the maize pathogens *Sporisorium reiliana* and *Ustilago maydis* by dot blot hybridization and PCR-based assays. Plant Dis. 83, 390–395. doi: 10.1094/PDIS.1999.83.4.390 30845593

[B220] Yadava P. Abhishek A. Singh R. Singh I. Kaul T. Pattanayak A.. (2017). Advances in maize transformation technologies and development of transgenic maize. Front. Plant Sci. 7, 1949. doi: 10.3389/fpls.2016.01949 28111576 PMC5216042

[B221] Yamaguchi-Shinozaki K. Shinozaki K. (1994). A novel cis-acting element in an arabidopsis gene is involved in responsiveness to drought, low-temperature, or high-salt stress. Plant Cell 6 (2), 251–264. doi: 10.1105/tpc.6.2.251 8148648 PMC160431

[B222] Yang J. Kloepper J. W. Ryu C. M. (2009). Rhizosphere bacteria help plants tolerate abiotic stress. Trends Plant Sci. 14, 1–4. doi: 10.1016/j.tplants.2008.10.004 19056309

[B223] Ying H. Xue Y. Yan K. Wang Y. Yin Y. Liu Z.. (2020). Safeguarding food supply and groundwater safety for maize production in china. Environ. Sci. Technol. 54 (16), 9939–9948. doi: 10.1021/acs.est.9b05642 32706248

[B224] Yun P. Xu L. (2018). *Piriformospora indica* improves salinity stress tolerance in *Zea mays* L. plants by regulating Na+ and K+ loading in root and allocating K+ in shoot. Plant Growth Regul. 86, 2. doi: 10.1007/s10725-018-0431-3

[B225] Yuriko O. Keishi O. Kazuo S. Tran L. (2014). Response of plants to water stress. Front. Plant Sci. 5, 86. doi: 10.3389/fpls.2014.00086 24659993 PMC3952189

[B226] Zaidi P. H. Maniselvan P. Srivastava A. Yadav P. Singh R. P. (2010). Genetic analysis of water-logging tolerance in tropical maize (*Zea mays* L.). Maydica 55, 17–26.

[B227] Zaidi P. H. Vinayan M. T. Nair S. K. (2023). Heat-tolerant maize for rainfed hot, dry environments in the lowland tropics: From breeding to improved seed delivery. Crop J. 4, 986–1000. doi: 10.1016/j.cj.2023.06.008

[B228] Zandalinas S. I. Mittler R. Balfagón D. Arbona V. Gómez-Cadenas A. (2018). Plant adaptations to the combination of drought and high temperatures. Physiol. plant. 162, 2–12. doi: 10.1111/ppl.12540 28042678

[B229] Zhang J. Wang L. H. Yang J. C. Liu H. Dai J. L. (2015). Health risk to residents and stimulation to inherent bacteria of various heavy metals in soil. Sci. total Environ. 508, 29–36. doi: 10.1016/j.scitotenv.2014.11.064 25437950

[B230] Zhang W. Deng S. Zhao Y. Xu W. Liu Q. Zhang Y.. (2021). qMrdd2, a novel quantitative resistance locus for maize rough dwarf disease. BMC Plant Biol. 21, 307. doi: 10.1186/s12870-021-03107-1 34193031 PMC8244169

[B231] Zhang X. Huang C. Meng Y. Liu X. Gao Y. Liu Z.. (2023). Physiological mechanism of waterlogging stress on yield of waxy maize at the jointing stage. Plants 12, 3034. doi: 10.3390/plants12173034 37687280 PMC10489971

[B232] Zorb C. Herbst R. Forreiter C. Schubert S. (2009). Short-term effects of salt exposure on the maize chloroplast protein pattern. Proteomics 9, 4209–4220. doi: 10.1002/pmic.200800791 19688749

